# Green Oxidation of Heterocyclic Ketones with Oxone in Water

**DOI:** 10.1021/acs.joc.3c01513

**Published:** 2023-10-12

**Authors:** Alessandro Giraudo, Edoardo Armano, Camillo Morano, Marco Pallavicini, Cristiano Bolchi

**Affiliations:** Dipartimento di Scienze Farmaceutiche, Università degli Studi di Milano, via Mangiagalli 25, I-20133, Milano, Italy

## Abstract

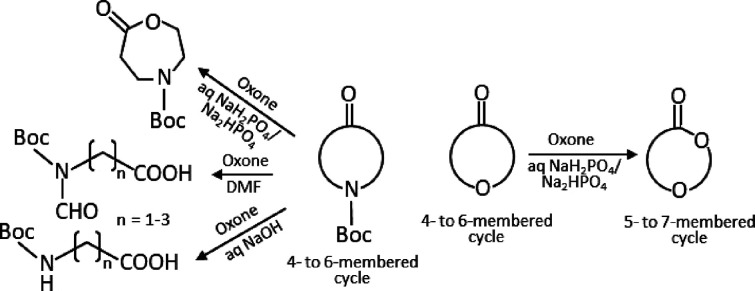

The recently reported efficient conversion of cyclic ketones to lactones by Oxone in neutral buffered water is extended to heterocyclic ketones, namely, cyclic *N*-Boc azaketones and oxoethers with the aim of obtaining *N*-protected azalactones and their analogues with oxygen in place of nitrogen. *N*-Boc-4-piperidinone and all the cyclic oxoethers were successfully oxidized to lactones, while the azacyclic ketones with nitrogen α-positioned to carbonyl were univocally transformed into *N*-Boc-ω-amino acids and *N*-Boc-*N*-formyl-ω-amino acids operating in alkaline water and DMF, respectively.

Expanding the use and finding new applications of easy to handle, nontoxic, and nonpollutant oxidizing agents are current goals of chemistry research that aim to combine efficiency with sustainability. Within the wide arsenal of oxidants, great attention has been directed to trichloroisocyanuric acid^1^ and Oxone,^2^ which meet these criterions and share synthetic applications, as exemplified by recently reported procedures of indoles oxidation^3^ and synthesis of benzo[*b*]chalcogenophenes.^4^ According to such approaches, we have latterly proposed a new protocol of Baeyer–Villiger (BV) oxidation of a series of ketones to lactones with Oxone in 1 M NaH_2_PO_4_/Na_2_HPO_4_ water solution (pH 7).^5^ Oxone (KHSO_5_-1/2KHSO_4_-1/2K_2_SO_4_, MW 307) is a green, cheap, and safe oxidant, which generates K_2_SO_4_ as the only byproduct. Strong acidity, due to the KHSO_4_ component, and high water solubility are its peculiar characteristics, which can be helpful and advantageous for some substrates and products, but contra-indicated for others requiring measures to be adopted for the oxidation feasibility such as water replacement with ionic liquids,^6^ use of biphasic systems in the presence of PTCs^7^ or of Oxone deposited on solid supports in apolar solvents^8^ or, more simply, as we have found, buffering of the water reaction environment to neutrality.^5^ Applied to eight cyclic ketones, our procedure has allowed lactones that are very important synthons such as γ-butyrolactone, δ-valerolactone, and ε-caprolactone to be easily and efficiently obtained with no hydrolysis.^5^

As a continuation of this research effort and in order to expand the scope of the method, we decided to study the BV oxidation of other substrates with Oxone under the conditions previously developed for cyclic ketones. We considered four *N*-Boc protected cyclic 3- and 4-oxo-amines, namely *N*-Boc-3-azetidinone (**1**), *N*-Boc-3-pyrrolidinone (**2**), *N*-Boc-3-piperidinone (**3**), and *N*-Boc-4-piperidinone (**4**), and the corresponding cyclic 3- and 4-oxo-ethers, namely 3-oxetanone (**5**), 3-oxo-tetrahydrofuran (**6**), tetrahydropyran-3-one (**7**), and tetrahydropyran-4-one (**8**) (Figure 1).

**Figure 1:**
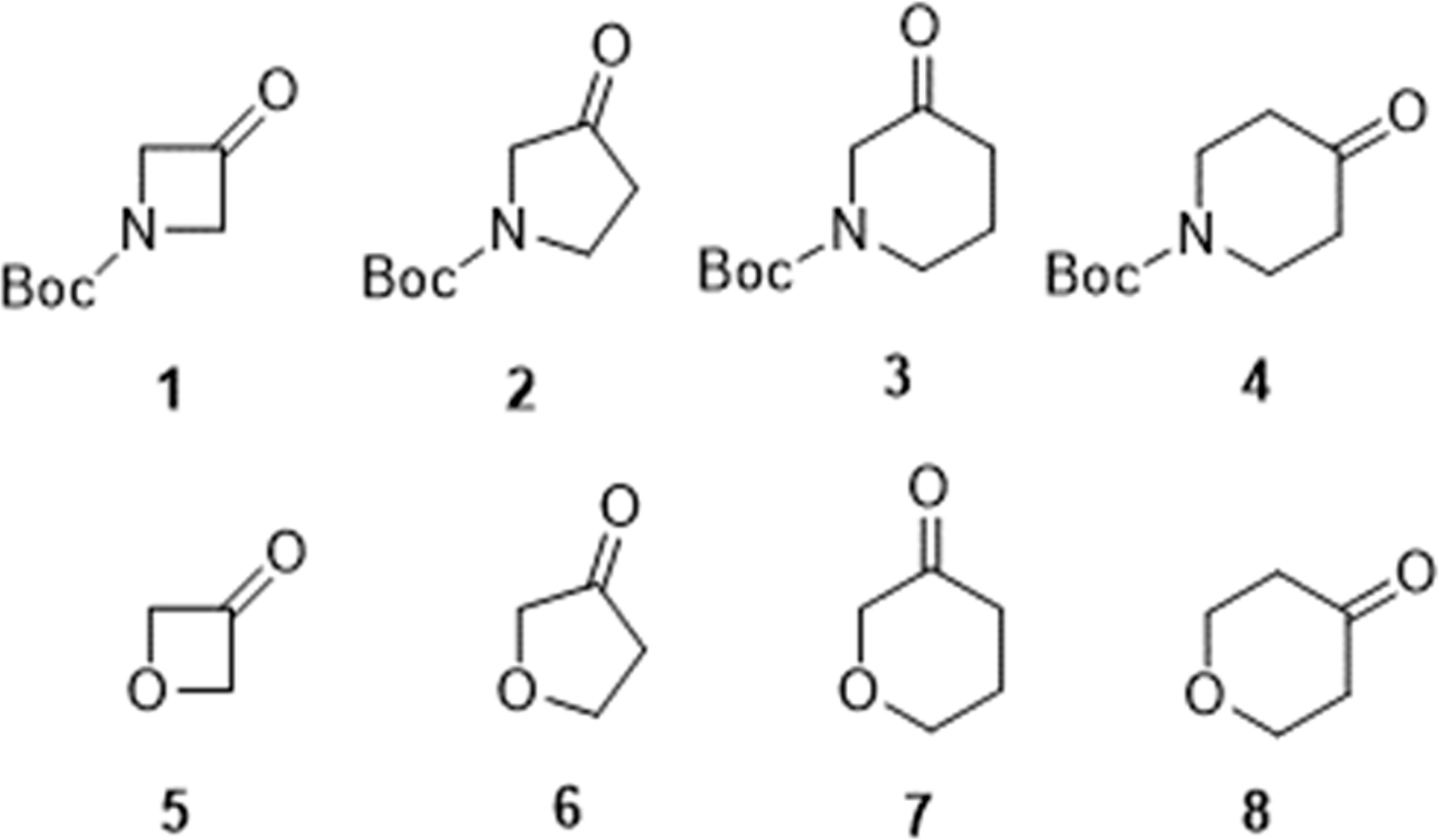
Heterocyclic ketones submitted to oxidation with Oxone.

The choice of the cyclic oxo-amines, all commercially available as *N*-Boc derivatives, was inspired by the interest in azalactones that could result, in principle, from their BV oxidation (Figure 2) and be useful for polymerization to poly(aminoester)s, which are pH-responsive cationic polymers. Recently, the ring-expansion of **4** to *N*-Boc-4-azacaprolactone (**9**) by BV oxidation with *m*-chloroperoxybenzoic acid (MCPBA) in DCM and the subsequent ring-opening polymerization of the azalactone to poly(β-aminoester) have been reported.^9^ Instead, the ring expansions of **1**–**3** to azalactones by BV oxidation are not described. Indeed, preparations of *N*-Boc-3-azabutyrolactone and *N*-Boc-4-azavalerolactone are reported, but not by BV oxidation of **1** and **2** respectively, while *N*-Boc-3-azavalerolactone and *N*-Boc-3- and *N*-Boc-5-azacaprolactone are not described (Figure 2).

**Figure 2:**
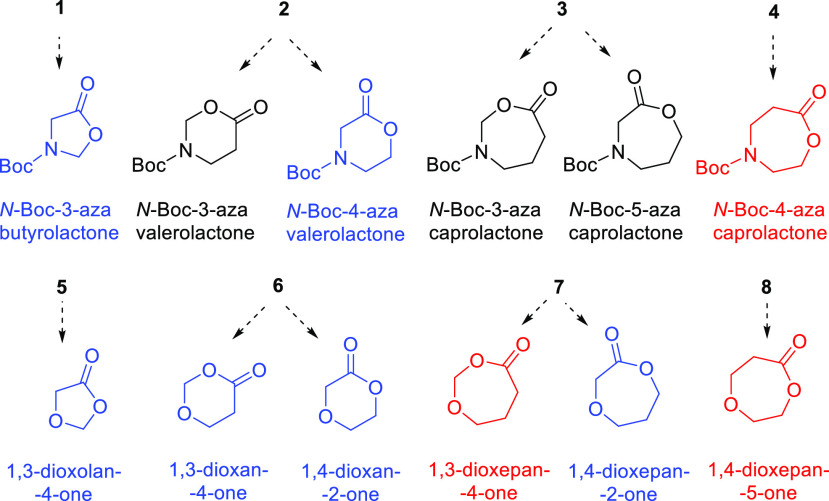
Lactones that may be obtained in principle by the oxidative ring expansion of **1**–**8**. In red, those reported in the literature as BV oxidation products of **4**, **7**, and **8**; in blue, those reported in the literature as resultant from other preparative procedures; and in black, those not described.

The choice of cyclic oxo-ethers **5**–**8** exactly paralleled that of cyclic oxo-amines **1**–**4** in order to make a comparison between the BV reactivity and regioselectivity displayed by the two classes of substrates and the stability of the products under the selected reaction conditions. Among the chosen cyclic oxo-ethers, as shown in Figure 2, the BV ring expansion is exemplified in literature for tetrahydropyran-3-one (**7**)^10^ and tetrahydropyran-4-one (**8**).^11^ Analogously to azalactones, some lactones derivable from cyclic oxo-ethers are useful monomers to develop degradable synthetic homo- and copolymers, such as poly(hemiacetal-ester)s and poly(ether-ester)s.^12^

We started from the conversion of **4** into **9**, which has been recently accomplished under classical BV conditions (MCPBA in DCM) in 73% yield,^9^ to have immediate feedback on the performance of our procedure based on the use of Oxone in neutral water environment. After 24 h of reaction at room temperature and standard workup, we isolated **9** by flash chromatography with 75% yield, in line with the literature datum and in confirmation of the stability of the ester function we had previously observed for the corresponding carba-lactone (Scheme 1).^5^

**Scheme 1:**
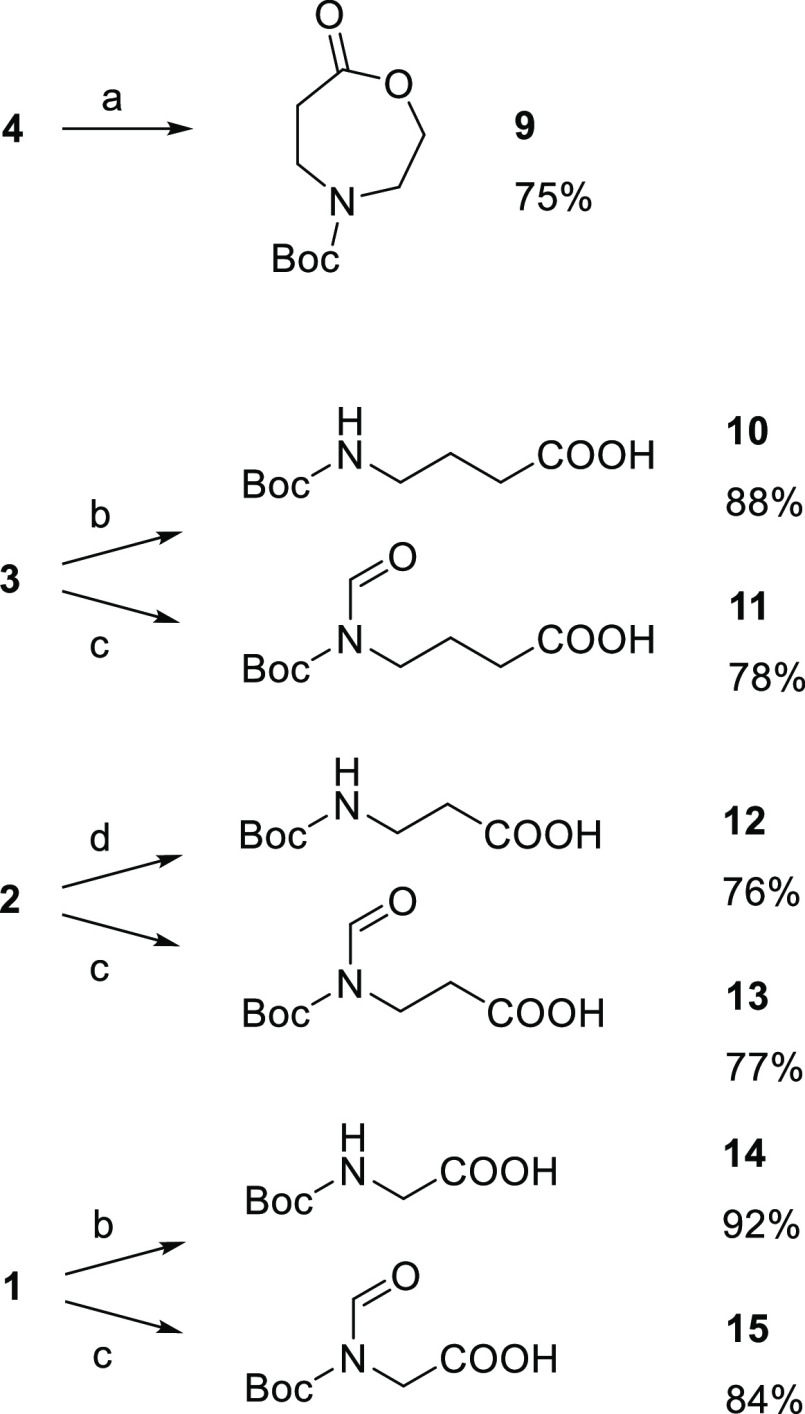
Oxidations of *N*-Boc Azacyclic Ketones 1-4 with Oxone General reaction conditions: (a) ketone (1 mmol), Oxone (8 mmol), 2 M NaH_2_PO_4_/Na_2_HPO_4_ water solution (pH = 7) (14 mL), room temperature, 24 h; (b) ketone (1 mmol), Oxone (2 mmol), 1 M NaOH (8 mL), room temperature, 30 min; (c) ketone (1 mmol), Oxone (2 mmol), DMF (5–9 mL), room temperature (**3** and **2**) or 60 °C (**1**), 1 h (**3**) or 16 h (**2**) or 5 h (**1**); (d) (1) ketone (1 mmol), Oxone (2 mmol), 2 M NaH_2_PO_4_/Na_2_HPO_4_ water solution (pH = 7) (9 mL), room temperature, 2 h, and (2) 1 M NaOH (9 mL), room temperature, 30 min.

Afterward, we considered substrate **3**, which is a positional isomer of **4** (Scheme 1). For this substrate, we did not observe univocal conversion to lactone as for **4**. Within the first hour of reaction, *N*-Boc-3-azacaprolactone, one of the two regioisomeric azalactones obtainable from **3** (Figure 2), was the main product (60–75%) flanked by minor quantities of *N*-Boc-γ-aminobutyric acid (**10**). This product became predominant in overnight or 48 h reactions or by increasing the reaction temperature to 40 °C. After 1 h of reaction at room temperature, we detected also a third product, *N*-Boc-*N*-formyl-γ-aminobutyric acid (**11**), whose quantity became close to that of **10** in a 24 h reaction, when *N*-Boc-3-azacaprolactone was reduced to about 10%. Anyway, regardless of when the reaction was stopped, *N*-Boc-3-azacaprolactone was chromatographically isolated with poor yield and always in mixture with a nonnegligible amount (>10%) of **10**, which increased during storage. The intrinsic instability of the azalactone, reasonably due to the hemiaminal ether substructure, and, on the other hand, our interest in simple Oxone BV oxidation protocols leading to a unitary product prompted us to develop reaction conditions selecting the other two products, namely, **10** and **11**. The reaction with Oxone in phosphate buffer did not allow product selectivity, while that in 1 M NaOH quantitatively provided **10** in 30 min at room temperature. High-yield conversion of **3** into **11** was instead accomplished with Oxone in DMF in 1 h at room temperature (Scheme 1).

The same two transformations were analogously performed starting from **1** to give, respectively, *N*-Boc-glycine (**14**) and *N*-Boc-*N*-formylglycine (**15**). Otherwise, to efficiently convert **2** into *N*-Boc-β-alanine (**12**), it was necessary to oxidize the substrate with Oxone in phosphate buffer and to treat the resultant mixture of oxidation products with 1 M NaOH at room temperature for 30 min, while the oxidation to *N*-formyl-*N*-Boc-β-alanine (**13**) was performed similarly as with **1** and **3** (Scheme 1). In literature, preparations of **11** and **13** have been reported through ruthenium tetroxide oxidative cleavage of the *endo*-cyclic carbon–carbon double bond of *N*-Boc-1,2,3,4-tetrahydropyridine and *N*-Boc-2-pyrroline, respectively.^13^

On the basis of the outcome of the oxidations of the cyclic oxo-amines **1**–**4**, we turned to the corresponding cyclic oxo-ethers **5**–**8** prefiguring analogous scenarios of different reactivity and product stability in the presence of Oxone in a neutral water environment. On the contrary, we observed uniform behavior of the cyclic oxo-ethers (Scheme 2). Oxidation of the three cyclic oxo-ethers **5**, **6**, and **7**, accomplished at 0 °C, proceeded as that of **8** at room temperature, without the differences previously observed between **1**–**3** and **4**. All four substrates were quantitatively BV oxidized to lactones, and the four crude lactones, including the three lactone acetals (**17**–**19**) resulting from **5**–**7**, were stable enough to be chromatographically purified. The two unsymmetrically substituted ketones **6** and **7** displayed the same BV regioselectivity with preferential migration of the O-linked methylene resulting in the formation of the lactone acetals **17** and **18**, according to what is reported in the literature for **7**(10) and more complex molecules containing **6** as a substructure.^14^

**Scheme 2:**
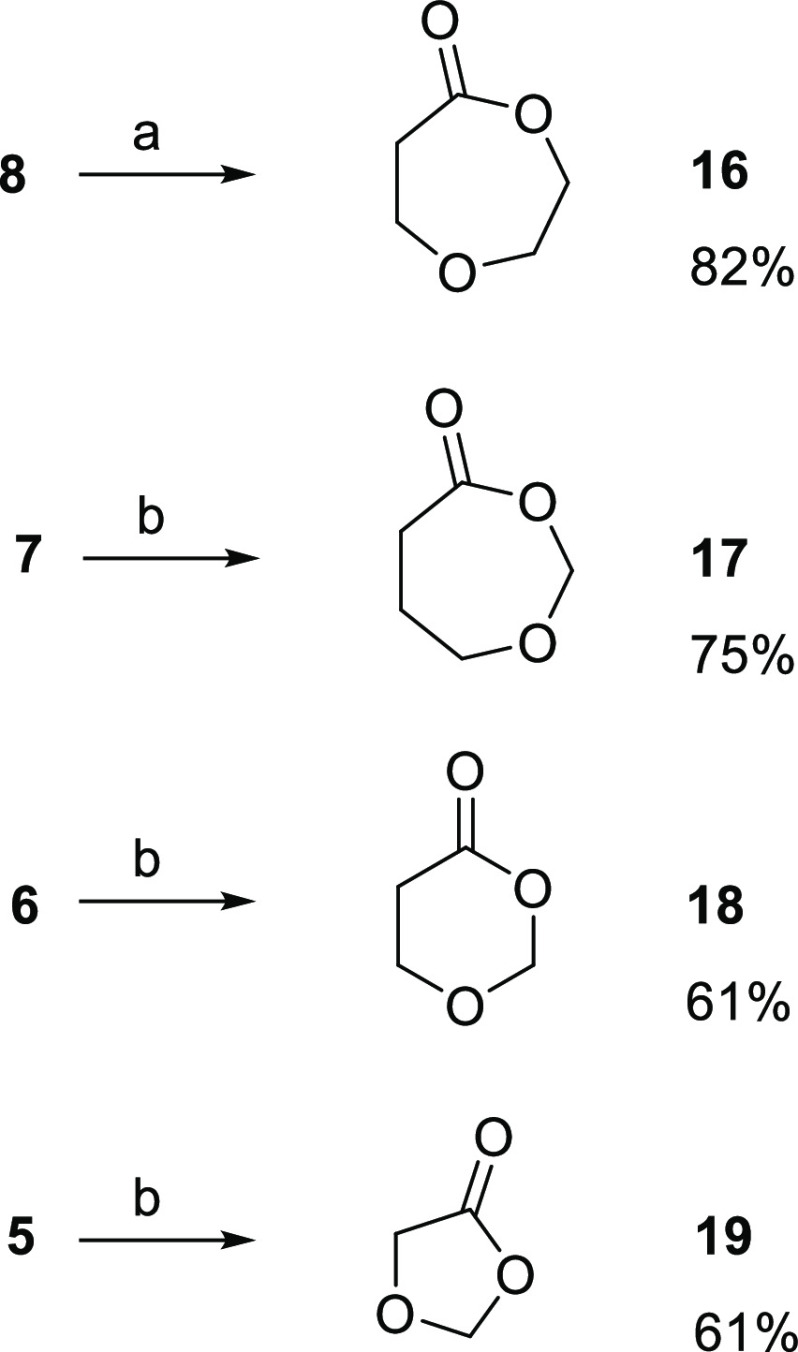
Oxidations of Cyclic Oxo-ethers **5**–**8** with Oxone General reaction conditions: (a) ketone (1 mmol), Oxone (4 mmol), 1 M NaH_2_PO_4_/Na_2_HPO_4_ water solution (pH = 7) (4 mL), room temperature, 40 min; (b) ketone (1 mmol), Oxone (2 mmol), 2 M NaH_2_PO_4_/Na_2_HPO_4_ water solution (pH = 7) (4 mL), 0 °C, 10 min (**7** and **6**) or 3 min (**5**).

Of the four lactones here obtained from **5**–**8** by BV oxidation, only **16** and **17** have been previously reported as a product resulting from BV oxidation of **8**(11) and **7**,^10^ respectively. Recently, the BV oxidation of **8** to **16** has been accomplished with 2,2′-diperoxydiphenic acid in DCM^11c^ and that of **7** to **17** with MCPBA in DCM.^10^ In this latter case, the authors observed the formation of an impurity produced by the reaction of **17** with *m*-chlorobenzoic acid and the autopolymerization and decomposition of **17** caused by the attempts to remove such an impurity. The preparations of **18** and **19** have been described in the literature, but not from **6** and **5**.^15,16^

In conclusion, we have widened the application of a green BV oxidation procedure, based on the use of Oxone in water, previously developed to convert cyclic ketones into lactones, considering a series of heterocyclic ketones, with α- or β-positioned heteroatoms, as new substrates. Our investigation demonstrates that cyclic ketones with an intraannular oxygen, whether α- or β-positioned, are easily oxidized to lactones, exclusively yielding, when the ethereal oxygen is α-positioned, the lactone acetals. On the other hand, under analogous reaction conditions, *N*-Boc azacyclic ketones give different oxidation outcomes: lactonization, when nitrogen is β-positioned in the starting heterocyclic ketone, opening of the cycle to ω-amino acid and *N*-formyl ω-amino acid, when nitrogen is α-positioned. In the latter case, it is possible to direct the reaction to the exclusive formation of the *N*-Boc ω-amino acid or its *N*-formylated derivative accomplishing the oxidation with Oxone in dilute aqueous sodium hydroxide or DMF respectively.

## Experimental Section

### General Experimental Details

Heterocyclic ketones **1**–**8** were purchased from commercial sources. An oil bath was used as the heating source for reactions that required heating. Flash chromatography purifications were performed by using Sfär Silica D 60 μm cartridges. ^1^H NMR and ^13^C{^1^H}-NMR spectra were recorded in CDCl_3_ at 300 and 75 MHz respectively, with a Varian Mercury 300 Spectrometer and elaborated with Mnova software. Chemical shifts are reported in ppm relative to residual solvent as an internal standard. Melting points were determined by a Buchi Melting Point B-540 apparatus. Thin-layer chromatography (TLC) analyses were carried out on alumina sheets precoated with silica gel 60 F254. High Resolution Mass Spectra (HRMS) were acquired by direct infusion on a ThermoScientific Orbitrap Elite (Thermo Fisher Scientific, Waltham, MA, United States) operated in positive ElectroSpray Ionization (ESI^+^).

#### N-*tert*-Butyloxycarbonyl-4-azacaprolactone (**9**)^9^

*N-tert*-Butyloxycarbonyl-4-oxopiperidine (**4**, 0.5 g, 2.5 mmol) was added to a solution of Oxone (6.17 g, 20.1 mmol) in 2 M NaH_2_PO_4_/Na_2_HPO_4_ buffer (35 mL, pH 7). The reaction was stirred at room temperature for 24 h. The mixture was extracted with EtOAc (3 × 10 mL). The combined organic layers were dried (Na_2_SO_4_) and evaporated under reduced pressure. Purification by flash column chromatography (Petroleum ether/EtOAc 1:1) gave **9** as a white amorphous solid; 75% yield; *R*_f_ (Petroleum ether/EtOAc 1:1 stained with KMnO_4_) 0.38; ^1^H NMR (300 MHz, CDCl_3_) δ 4.30–4.21 (m, 2H), 3.84–3.72 (m, 2H), 3.72–3.58 (m, 2H), 2.85–2.75 (m, 2H), 1.47 (s, 9H); ^13^C{^1^H} NMR (75 MHz, CDCl_3_) δ 174.0, 154.4, 81.1, 69.5, 47.7, 41.2, 37.6, 28.4.

#### General Procedure for the Synthesis of Compounds **10** and **14**

The appropriate ketone (1 equiv) was added to a solution of Oxone (2 equiv) in 1 M NaOH. The reaction was stirred at room temperature for 30 min. The mixture was acidified with 1 M HCl and extracted with EtOAc (3 × 10 mL). The combined organic layers were dried in Na_2_SO_4_ and concentrated. Purification by flash column chromatography on silica gel (DCM/MeOH 95:5) gave the corresponding products.

#### N-tert-Butoxycarbonyl-γ-aminobutyric acid (**10**)^16^

Obtained from *N*-*tert*-butoxycarbonyl-3-piperidone (**3**, 1.0 g, 5.0 mmol) and Oxone (3.1 g, 10.0 mmol) in 1 M NaOH (40 mL) at room temperature for 30 min, following the general procedure reported above. Isolated as a yellow solid after column chromatography; 88% yield; *R*_f_ (DCM/MeOH 95:5 stained with KMnO_4_) 0.3; mp 49.2–52.5 °C; ^1^H NMR (300 MHz, CDCl_3_) δ 10.01 (br s, 1H), 6.10 (br s, 0.3H), 4.79 (br s, 0.7H), 3.26–3.01 (m, 2H), 2.36 (t, *J* = 7.2 Hz, 2H), 1.88–1.70 (m, 2H), 1.42 (s, 9H); ^13^C{^1^H} NMR (75 MHz, CDCl_3_) δ 178.4, 157.9 (minor rotamer), 156.3 (major rotamer), 81.0 (minor rotamer), 79.6 (major rotamer), 41.0 (minor rotamer), 39.9 (major rotamer), 31.4, 28.4, 25.2.

#### N-tert-Butoxycarbonyl-glycine (**14**)^17^

Obtained from *N*-*tert*-butoxycarbonyl-3-azetidinone (**1**, 0.2 g, 1.2 mmol) and Oxone (0.72 g, 2.34 mmol) in 1 M NaOH (10 mL) at room temperature for 30 min, following the general procedure reported above. Isolated as white solid after column chromatography; 92% yield; *R*_f_ (DCM/MeOH 95:5 stained with KMnO_4_) 0.16; mp 87–89.5 °C; ^1^H NMR (300 MHz, CDCl_3_) δ 8.87 (br s, 1H), 6.70 (br s, 0.4H), 5.05 (br s, 0.6H), 4.10–3.74 (m, 2H), 1.45 (s, 9H); ^13^C{^1^H} NMR (75 MHz, CDCl_3_) δ 174.5 (major rotamer), 174.0 (minor rotamer), 157.4 (minor rotamer), 156.2 (major rotamer), 81.9 (minor rotamer), 80.5 (major rotamer), 43.4 (minor rotamer), 42.3 (major rotamer), 28.3.

#### N-tert-Butoxycarbonyl-β-alanine (**12**)^18^

*N*-tert-Butoxycarbonyl-3-pyrrolidone (**2**, 0.2 g, 1.1 mmol) was added to a solution of Oxone (0.66 g, 2.2 mmol) in 2 M NaH_2_PO_4_/Na_2_HPO_4_ buffer (10 mL, pH 7). The reaction mixture was stirred at room temperature for 2 h and extracted with EtOAc (3 × 5 mL). The combined organic layers were dried over Na_2_SO_4_ and concentrated under reduced pressure to give a residue that was treated with 1 M NaOH (10 mL) under stirring at room temperature for 30 min. The mixture was acidified with 1 M HCl and extracted with EtOAc (3 × 5 mL). The combined organic layers were dried (Na_2_SO_4_) and concentrated. Purification of the residue by flash column chromatography (DCM/MeOH 97:3) afforded the title product as a white solid: 76% yield; *R*_f_ (DCM/MeOH 95:5 stained with KMnO_4_) 0.21; mp 73–75 °C; ^1^H NMR (300 MHz, CDCl_3_) δ 11.29 (br s, 1H), 6.28 (br s, 0.3H), 5.17 (br s, 0.7H), 3.52–3.14 (m, 2H), 2.74–2.33 (m, 2H), 1.41 (s, 9H); ^13^C{^1^H} NMR (75 MHz, CDCl_3_) δ 177.5 (major rotamer), 176.5 (minor rotamer), 157.7 (minor rotamer), 156.1 (major rotamer), 81.2 (minor rotamer), 79.8 (major rotamer), 37.3 (minor rotamer), 36.0 (major rotamer), 34.7, 28.4.

#### General Procedure for the Synthesis of Compounds **11**, **13**, and **15**

The appropriate ketone (1 equiv) was added to a suspension of Oxone (2–6 equiv) in DMF. The resulting suspension was stirred at the specified temperature. The mixture was quenched with H_2_O (10–20 mL) and then extracted with EtOAc (3 × 10–20 mL). The combined organic layers were dried in Na_2_SO_4_ and concentrated under reduced pressure. Purification by flash column chromatography on silica gel (DCM/MeOH 95:5) afforded the corresponding products.

#### N-tert-Butoxycarbonyl-N-formyl-γ-aminobutyric Acid (**11**)^13^

Obtained from *N*-*tert*-butoxycarbonyl-3-piperidone (**3**, 1.0 g, 5.0 mmol) and Oxone (3.1 g, 10.0 mmol) in DMF (25 mL) at room temperature for 1 h, following the general procedure reported above. Isolated as a yellow oil after column chromatography; 78% yield; *R*_*f*_ (DCM/MeOH 95:5 stained with KMnO_4_) 0.32; ^1^H NMR (300 MHz, CDCl_3_) δ 9.17 (s, 1H), 3.66 (t, *J* = 7.05 Hz 2H), 2.37 (t, *J* = 7.4 Hz, 2H), 1.96–1.78 (m, 2H), 1.54 (s, 9H); ^13^C{^1^H} NMR (75 MHz, CDCl_3_) δ 178.4, 163.4, 152.5, 84.4, 39.8, 31.3, 28.1, 23.3.

#### N-tert-Butoxycarbonyl-N-formyl-β-alanine (**13**)^13^

Obtained from *N-tert*-butoxycarbonyl-3-pyrrolidone (**2**, 0.2 g, 1.1 mmol) and Oxone (2.0 g, 6.5 mmol) in DMF (10 mL) at room temperature for 16 h, following the general procedure reported above. Isolated as a brown solid after column chromatography; 77% yield; *R*_*f*_ (DCM/MeOH 95:5 stained with KMnO_4_) 0.25; mp 68,2–70,3 °C; ^1^H NMR (300 MHz, CDCl_3_) δ 9.15 (s, 1H), 3.91 (t, *J* = 7.35 Hz, 2H), 2.61 (t, *J* = 7.35 Hz, 2H), 1.55 (s, 9H); ^13^C{^1^H} NMR (75 MHz, CDCl_3_) δ 177.0, 163.1, 152.1, 84.7, 36.2, 32.7, 28.1.

#### N-tert-Butoxycarbonyl-N-formylglycine (**15**)

Obtained from *N-tert*-butoxycarbonyl-3-azetidinone (**1**, 0.2 g, 1.17 mmol) and Oxone (2.16 g, 7.0 mmol) in DMF (10 mL) at 60 °C for 5 h, following the general procedure reported above. Isolated as a white solid after column chromatography; 84% yield; *R*_*f*_ (DCM/MeOH 95:5 stained with KMnO_4_) 0.19; mp 101,2–103,6 °C; ^1^H NMR (300 MHz, CDCl_3_) δ 9.20 (s, 1H), 4.39 (s, 2H), 1.54 (s, 9H); ^13^C{^1^H} NMR (75 MHz, CDCl_3_) δ 173.5, 162.6, 151.6, 85.3, 41.3, 28.0. HRMS (ESI^+^) *m*/*z* calcd. for C_8_H_13_NO_5_Na [M + Na]^+^ 226.06914, found 226.06833.

#### General Procedure for the Synthesis of Compounds **16**–**19**

The appropriate ketone (1 equiv) was added to a solution of Oxone (2–4 equiv) in NaH_2_PO_4_/Na_2_HPO_4_ buffer (1 or 2 M as specified, pH = 7) at room temperature or 0 °C. The solution was stirred 3–40 min. EtOAc (15–30 mL) was added, and the mixture was stirred vigorously. The phases were separated, and the water phase was extracted again with EtOAc (2 × 15–30 mL). The combined organic phases were washed with brine, dried over Na_2_SO_4_, and concentrated. Purification of the resultant residue by flash chromatography on silica gel (gradient of petroleum ether/EtOAc from 0% to 50% EtOAc) afforded the corresponding products.

#### 1,4-Dioxepan-5-one (**16**)^12d^

Obtained from tetrahydropyran-4-one (**8**, 0.5 mL, 5.4 mmol) and Oxone (6.66 g, 21.7 mmol) in 1 M NaH_2_PO_4_/Na_2_HPO_4_ buffer (21.7 mL) at room temperature for 40 min, following the general procedure reported above. Concentration of the organic extracts gave **16** (573 mg, 91.5%) as a unitary product with only traces of impurities (see ^1^H NMR spectrum of the crude), which was isolated as an amorphous solid by column chromatography with 82% final yield; *R*_f_ (Petroleum ether/EtOAc 6:4 stained with KMnO_4_) 0.19; ^1^H NMR (300 MHz, CDCl_3_) δ 4.33–4.24 (m, 2H), 3.94–3.84 (m, 2H), 3.84–3.77 (m, 2H), 2.93–2.83 (m, 2H); ^13^C{^1^H} NMR (75 MHz, CDCl_3_) δ 174.1, 70.7, 70.3, 64.7, 39.2.

#### 1,3-Dioxepan-4-one (**17**)^10^

Obtained from tetrahydropyran-4-one (**7**, 0.3 mL, 3.25 mmol) and Oxone (2.0 g, 6.5 mmol) in 2 M NaH_2_PO_4_/Na_2_HPO_4_ buffer (13 mL) at 0 °C for 10 min, following the general procedure reported above. Concentration of the organic extracts gave **17** (347 mg, 92%) as a unitary product with only traces of impurities (see ^1^H NMR spectrum of the crude), which was isolated as a colorless oil by column chromatography with 75% final yield; *R*_f_ (Petroleum ether/EtOAc 6:4 stained with KMnO_4_) 0.22; ^1^H NMR (300 MHz, CDCl_3_) δ 5.24 (s, 2H), 4.04–3.92 (m, 2H), 2.91–2.76 (m, 2H), 2.00–1.82 (m, 2H); ^13^C{^1^H} NMR (75 MHz, CDCl_3_) δ 174.2, 93.3, 73.5, 34.1, 24.8.

#### 1,3-Dioxan-4-one (**18**)^15^

Obtained from 3-oxo-tetrahydrofuran (**6**, 0.5 mL, 6.5 mmol) and Oxone (4.0 g, 13.0 mmol) in 2 M NaH_2_PO_4_/Na_2_HPO_4_ buffer (26 mL) at 0 °C for 10 min, following the general procedure reported above. Concentration of the organic extracts gave **18** (498 mg, 75%) as a unitary product with only traces of impurities (see ^1^H NMR spectrum of the crude), which was isolated as a colorless oil by column chromatography with a 61% final yield; *R*_f_ (Petroleum ether/EtOAc 6:4 stained with KMnO_4_) 0.19; ^1^H NMR (300 MHz, CDCl_3_) δ 5.38 (s, 2H), 4.10 (t, *J* = 6.6 Hz, 2H), 2.78 (t, *J* = 6.6 Hz, 2H); ^13^C{^1^H} NMR (75 MHz, CDCl_3_) δ 166.5, 94.1, 64.1, 31.0.

#### 1,3-Dioxolan-4-one (**19**)^12b^

Obtained from oxetan-3-one (**5**, 0.5 mL, 7.8 mmol) and Oxone (4.8 g, 15.6 mmol) in 2 M NaH_2_PO_4_/Na_2_HPO_4_ buffer (31.2 mL) at 0 °C for 3 min, following the general procedure reported above. Due to the high volatility of **19**, the organic extracts were concentrated to a 40% w/w residual content of ethyl acetate. The ^1^H NMR spectrum of the concentrated extracts (915 mg) showed the presence of **19** as a unique product, allowing calculation of an 80% yield. After column chromatography, **19** was isolated as a colorless oil with a 61% final yield; *R*_f_ (Petroleum ether/EtOAc 6:4 stained with KMnO_4_) 0.53; ^1^H NMR (300 MHz, CDCl_3_) δ 5.54 (s, 2H), 4.22 (s, 2H); ^13^C{^1^H} NMR (75 MHz, CDCl_3_) δ 171.3, 96.2, 62.6.

## Data Availability

The data underlying this study are available in the published article and its Supporting Information.

## Supporting Information Available

^1^H and ^13^C NMR spectra for the isolated products. (PDF)
