# Supplementary material for: Green Oxidation of Heterocyclic Ketones with Oxone in Water
Source: J Org Chem. 2023 Oct 12;88(21):15461–5. doi: 10.1021/acs.joc.3c01513 (PMC10629238; doi:10.1021/acs.joc.3c01513)

Green Oxidation of Heterocyclic Ketones with Oxone in Water

Alessandro Giraudo, Edoardo Armano, Camillo Morano, Marco Pallavicini, and Cristiano Bolchi*

Dipartimento di Scienze Farmaceutiche, Università degli Studi di Milano, via Mangiagalli 25, I-20133, Milano, Italy

Corresponding author: cristiano.bolchi@unimi.it

Supporting Information

Table of Contents

^{1}H NMR, ^{13}C NMR and HRMS spectra page 2-13

N-tert-Butyloxycarbonyl-4-azacaprolactone (9)

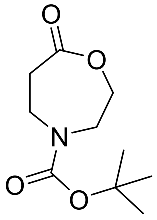

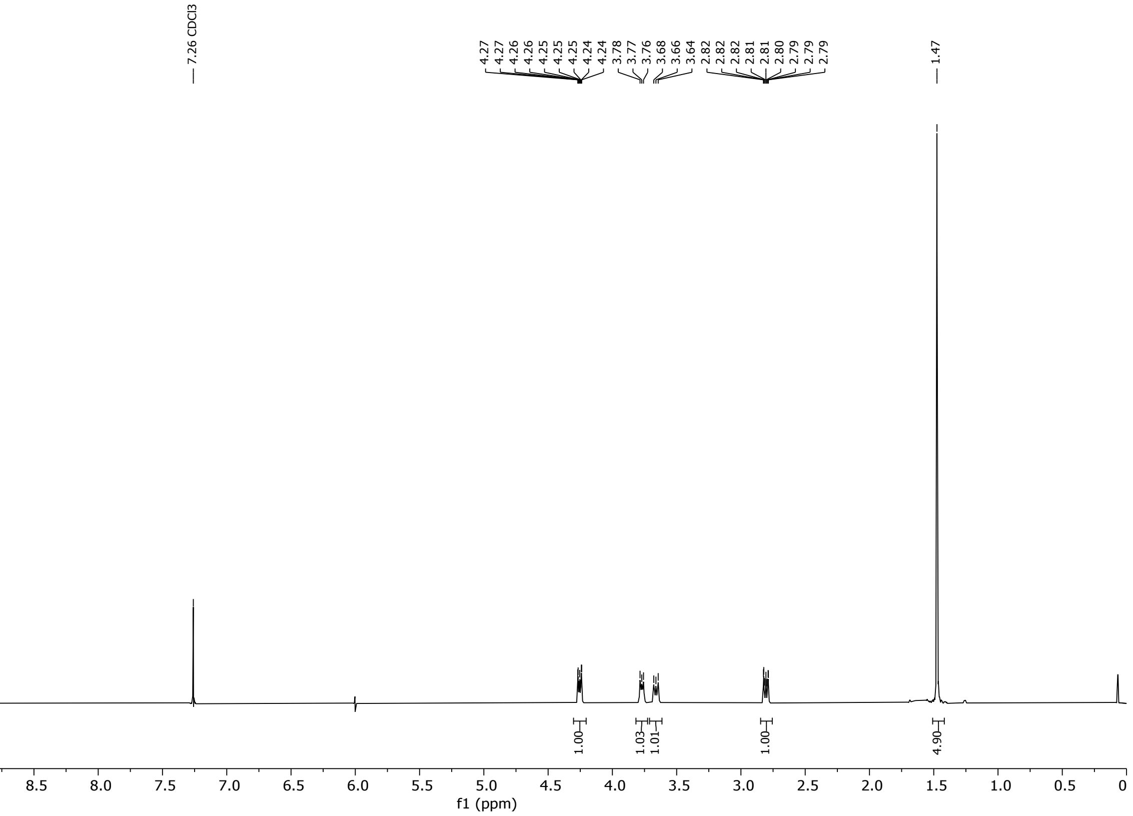

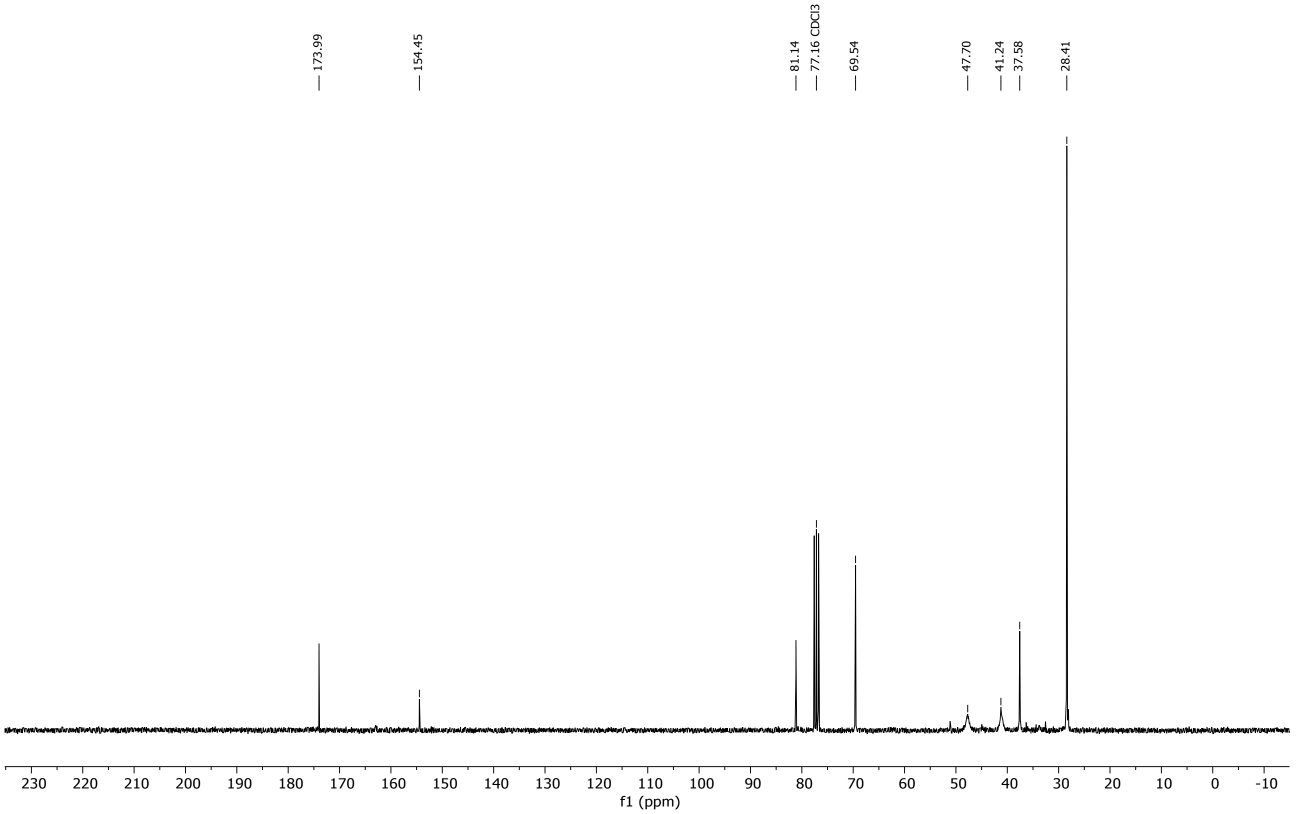

N-tert-Butyloxycarbonyl-γ-aminobutyric acid (10)

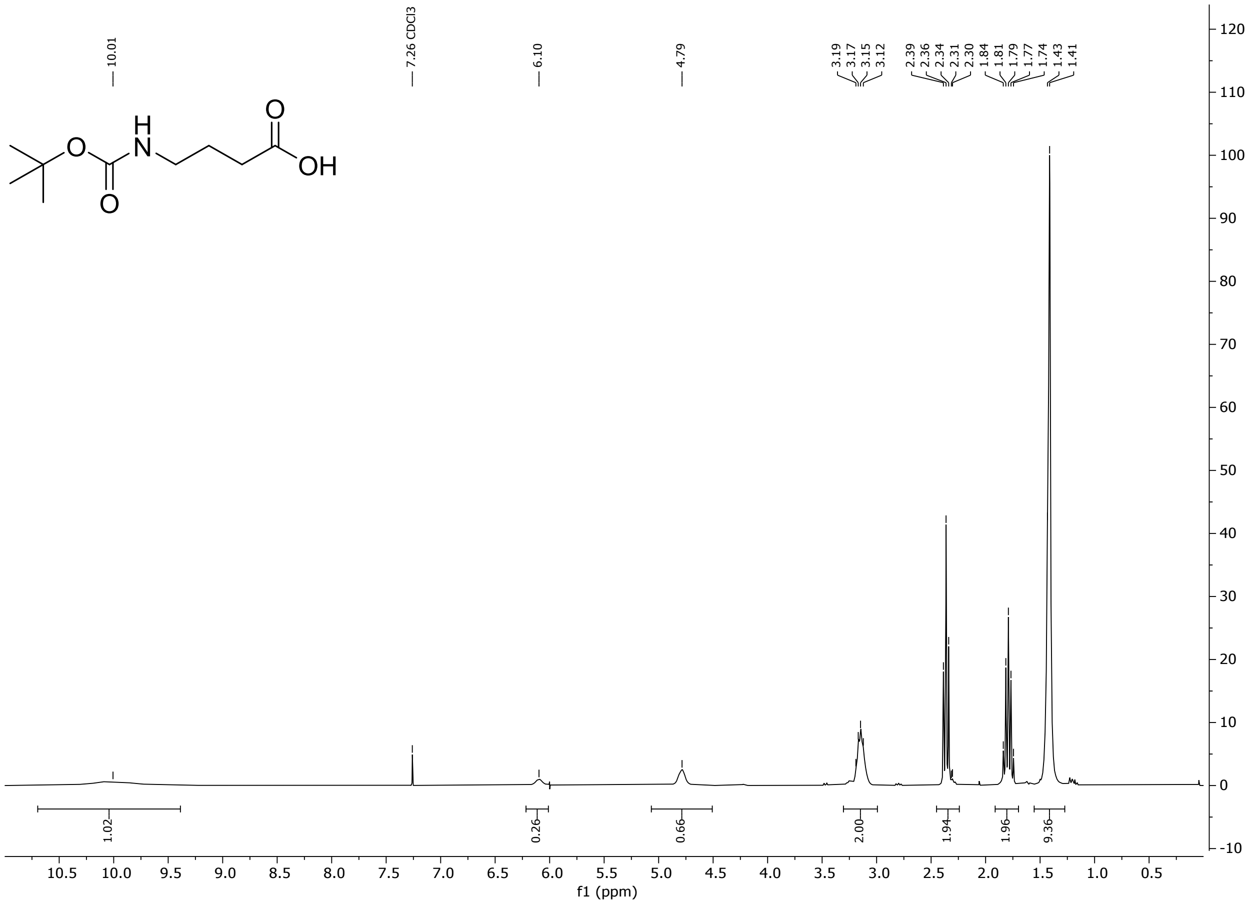

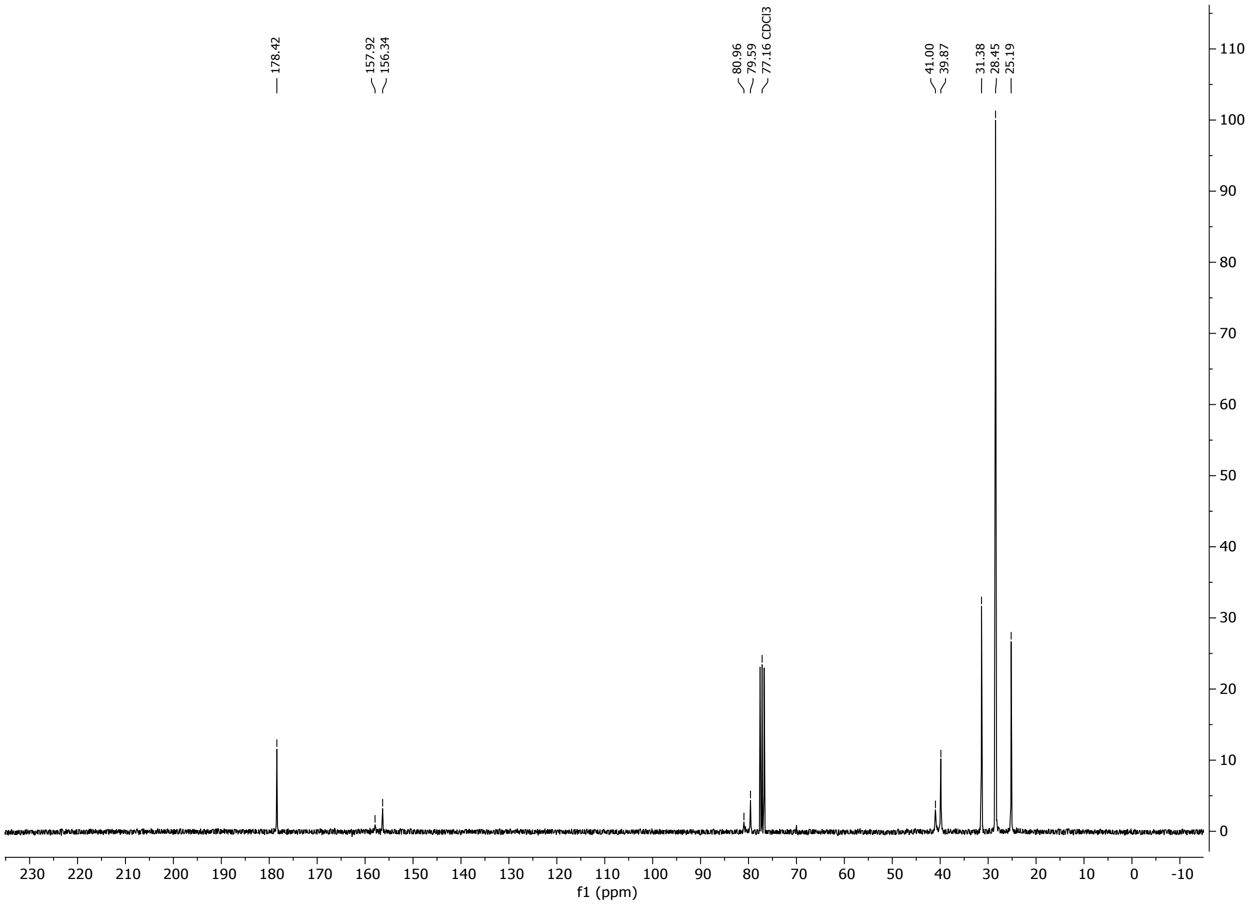

N-tert-Butyloxycarbonyl-N-formyl-γ-aminobutyric acid (11)

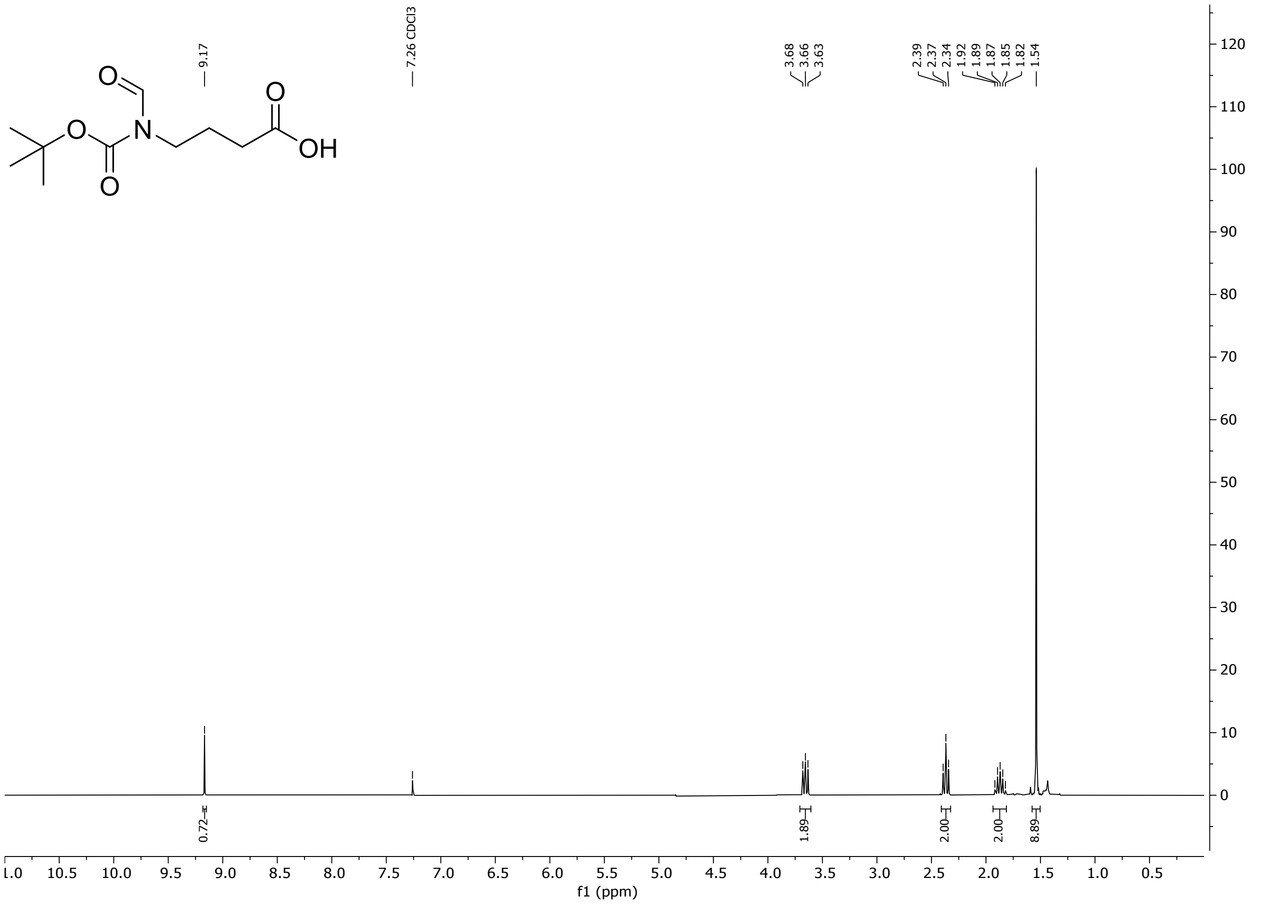

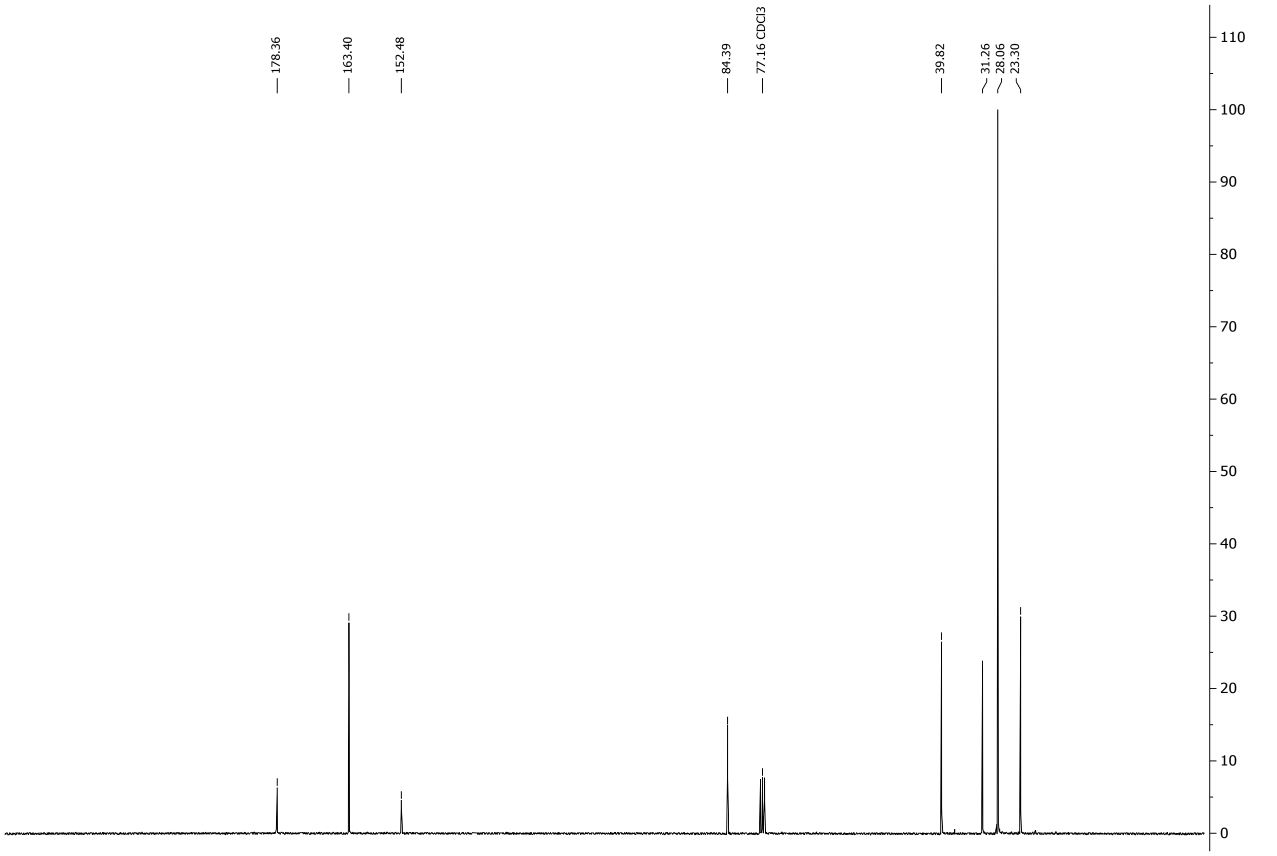

N-tert-Butyloxycarbonyl-β-alanine (12)

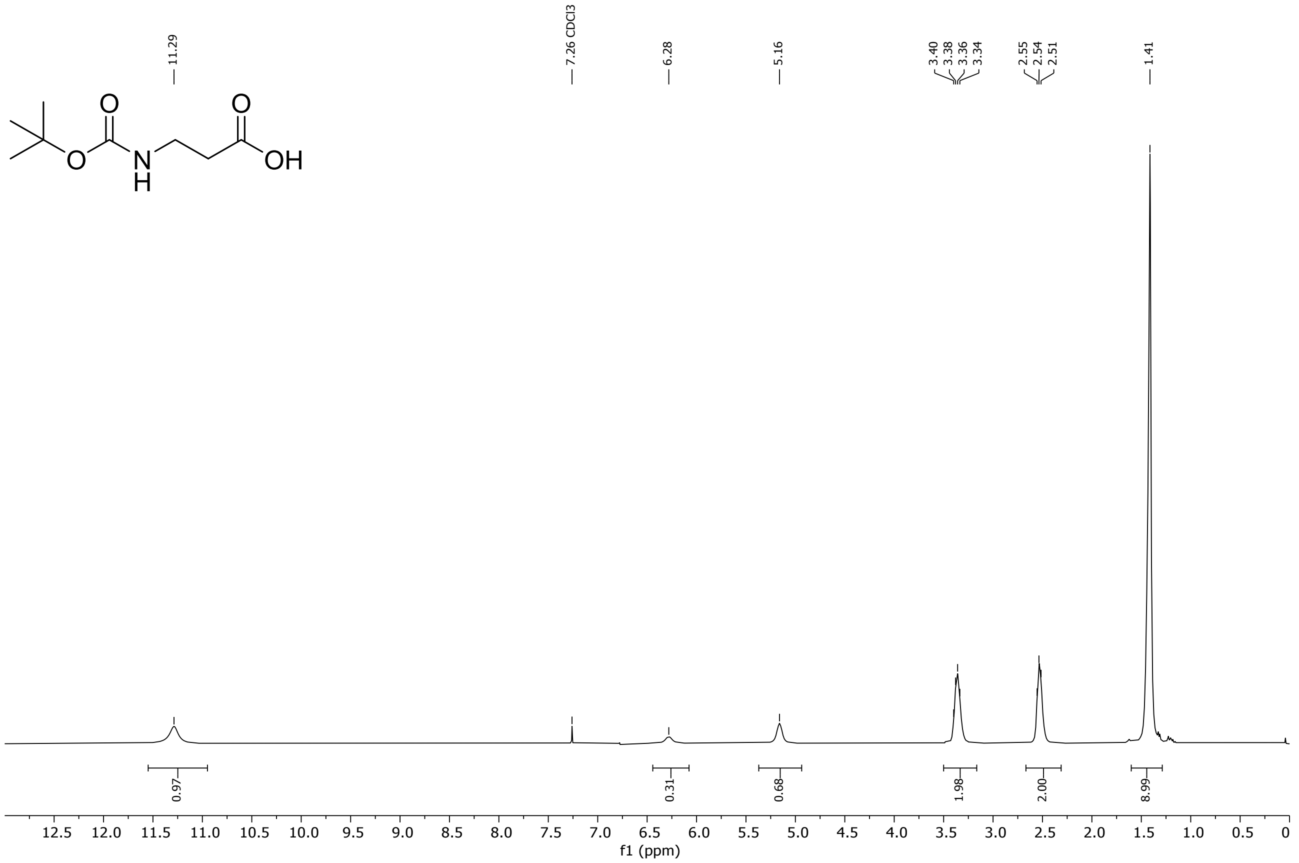

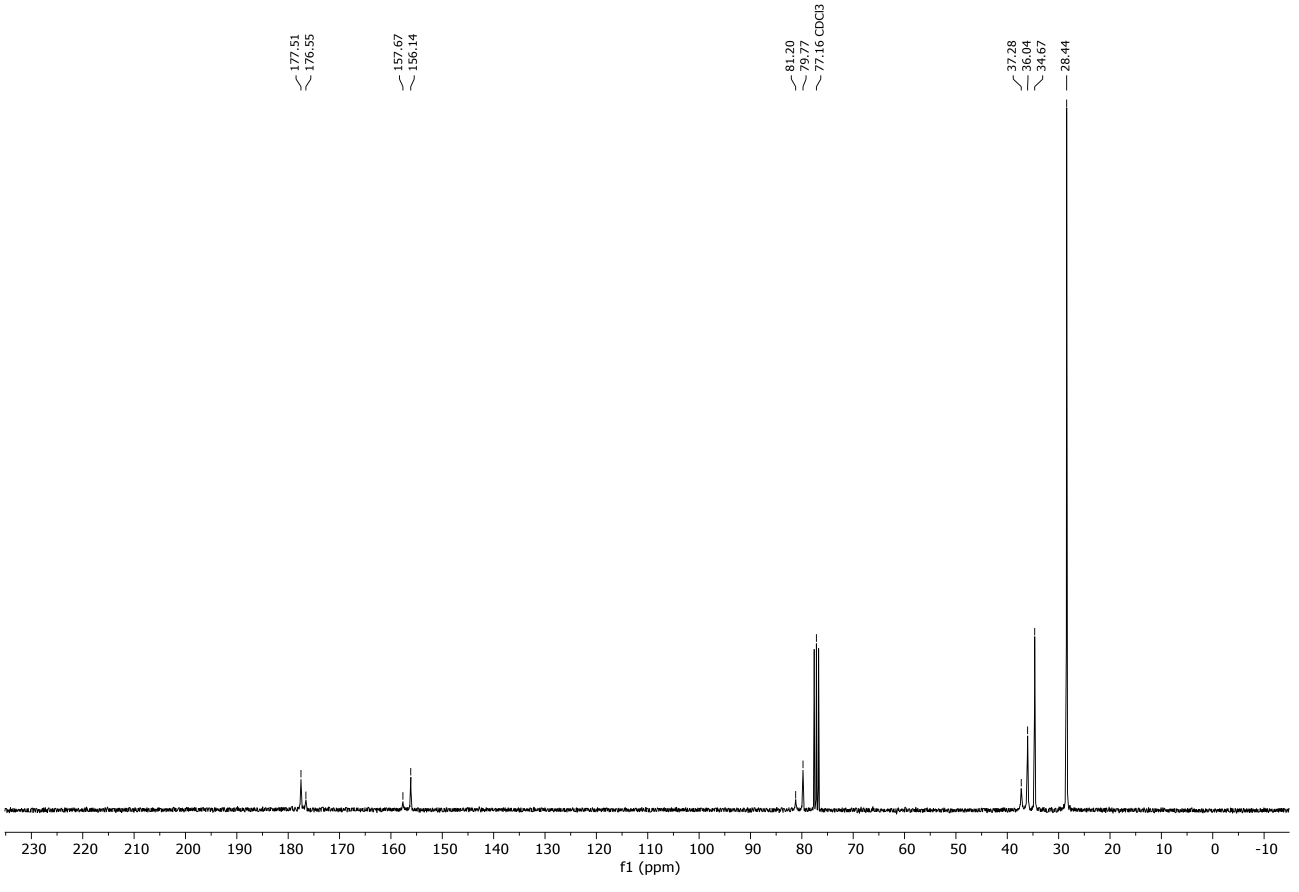

N-tert-Butyloxycarbonyl-N-formyl-β-alanine (13)

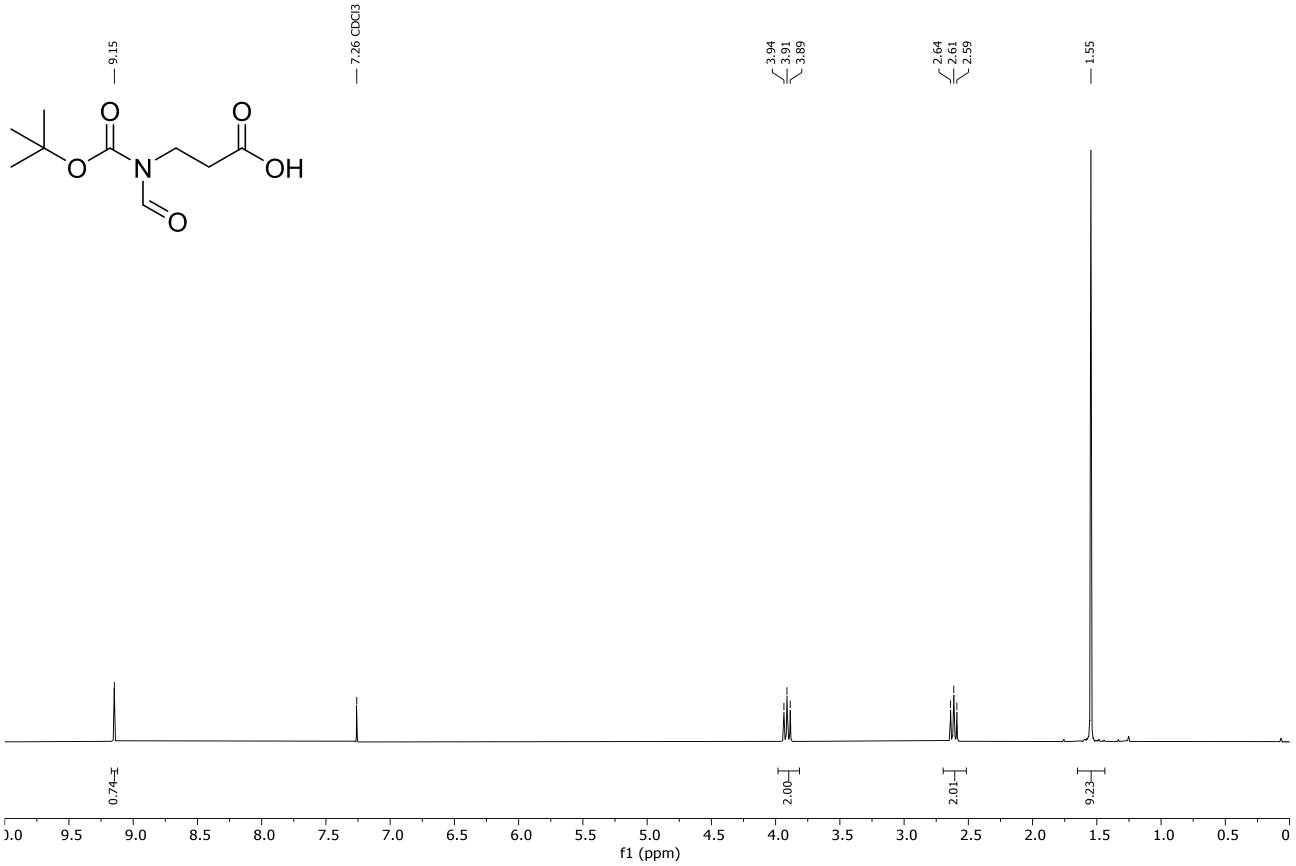

BL_8.8_m2_carbonio

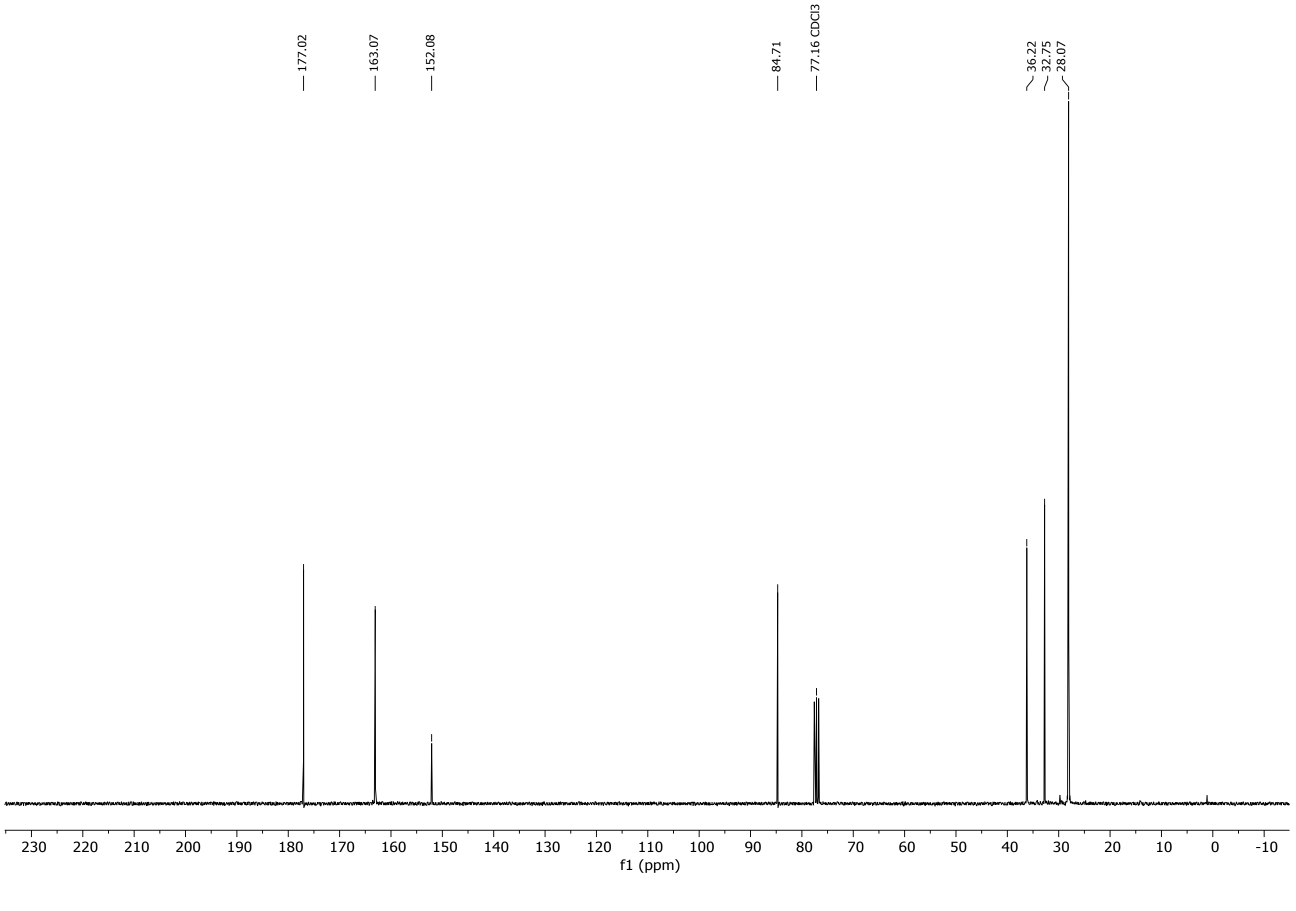

N-tert-Butyloxycarbonyl-glycine (14)

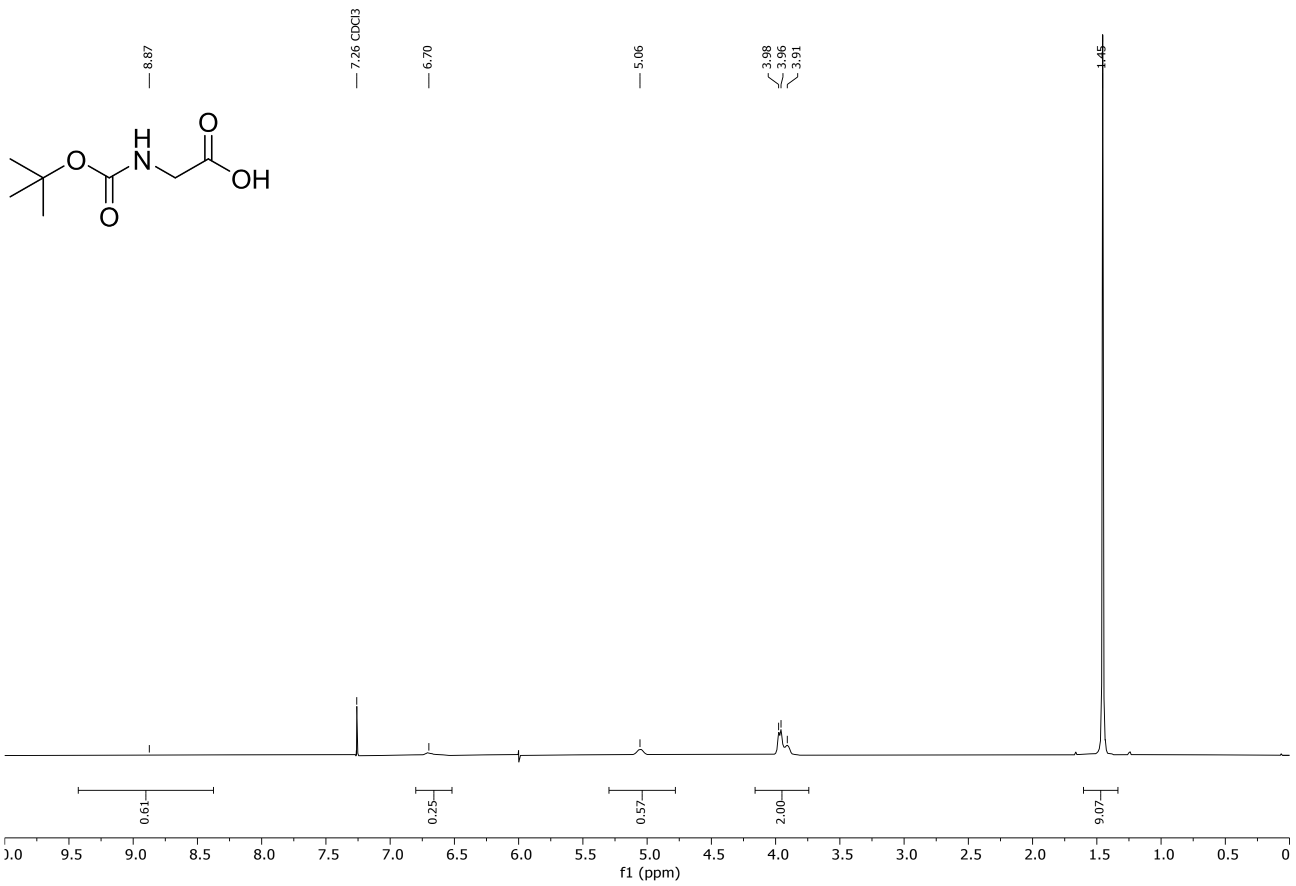

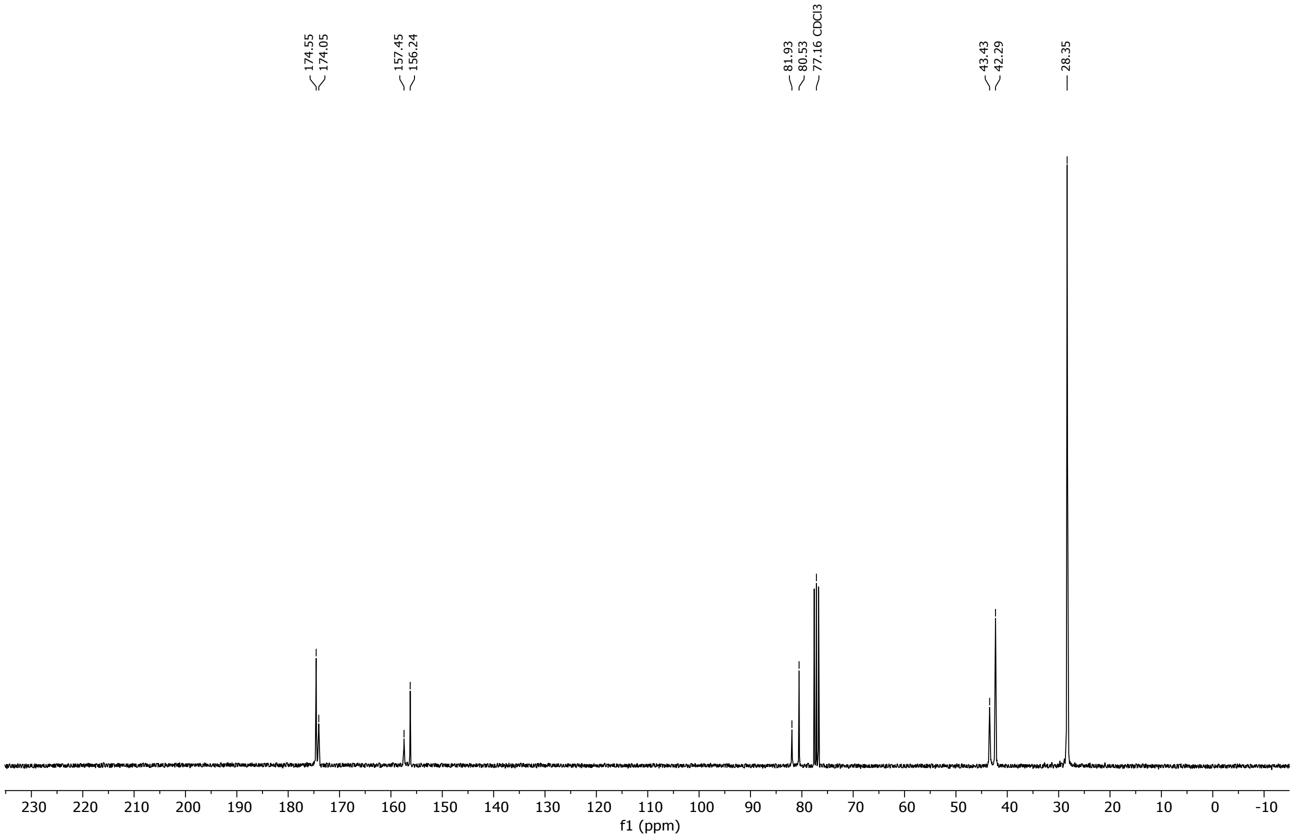

N-tert-Butoxycarbonyl-N-formyl-glycine (15)

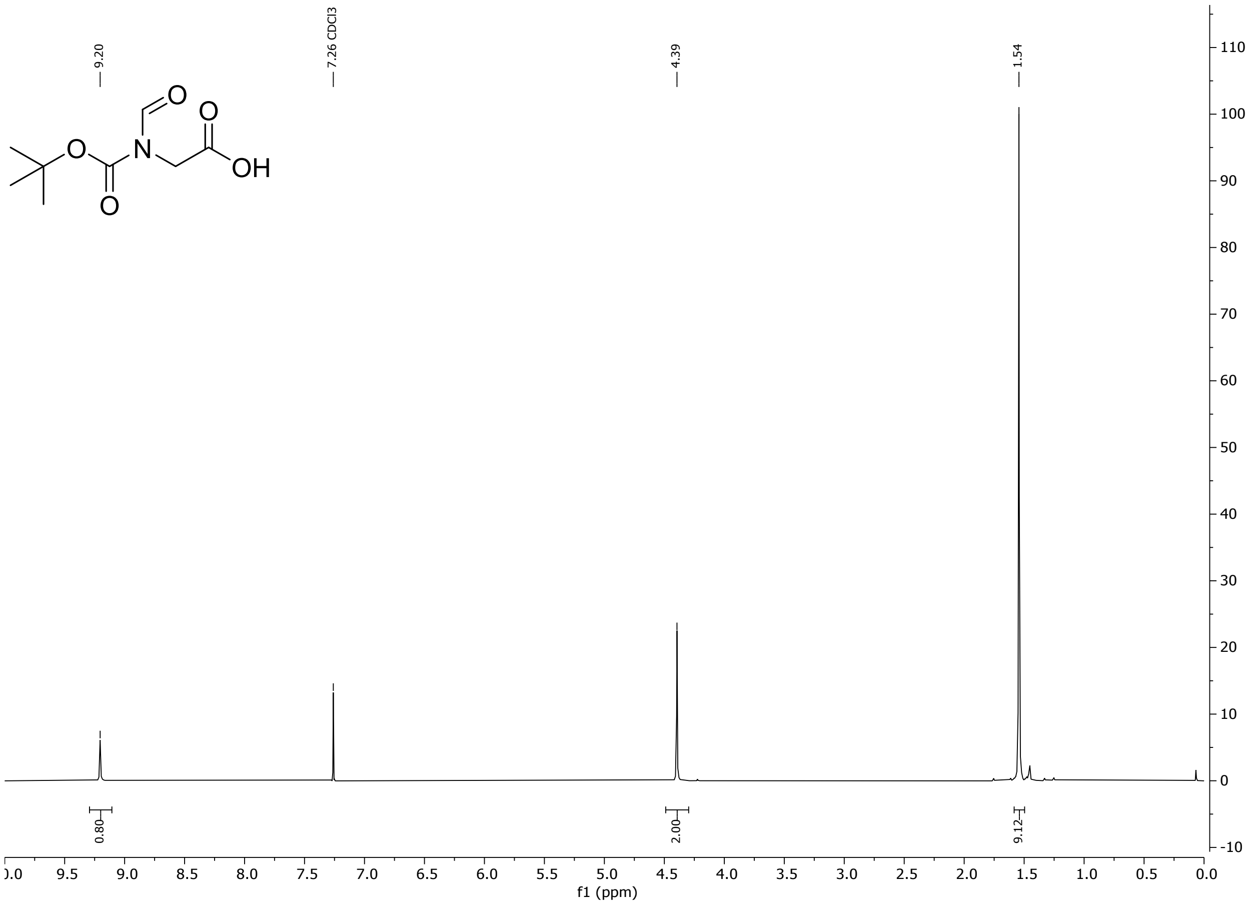

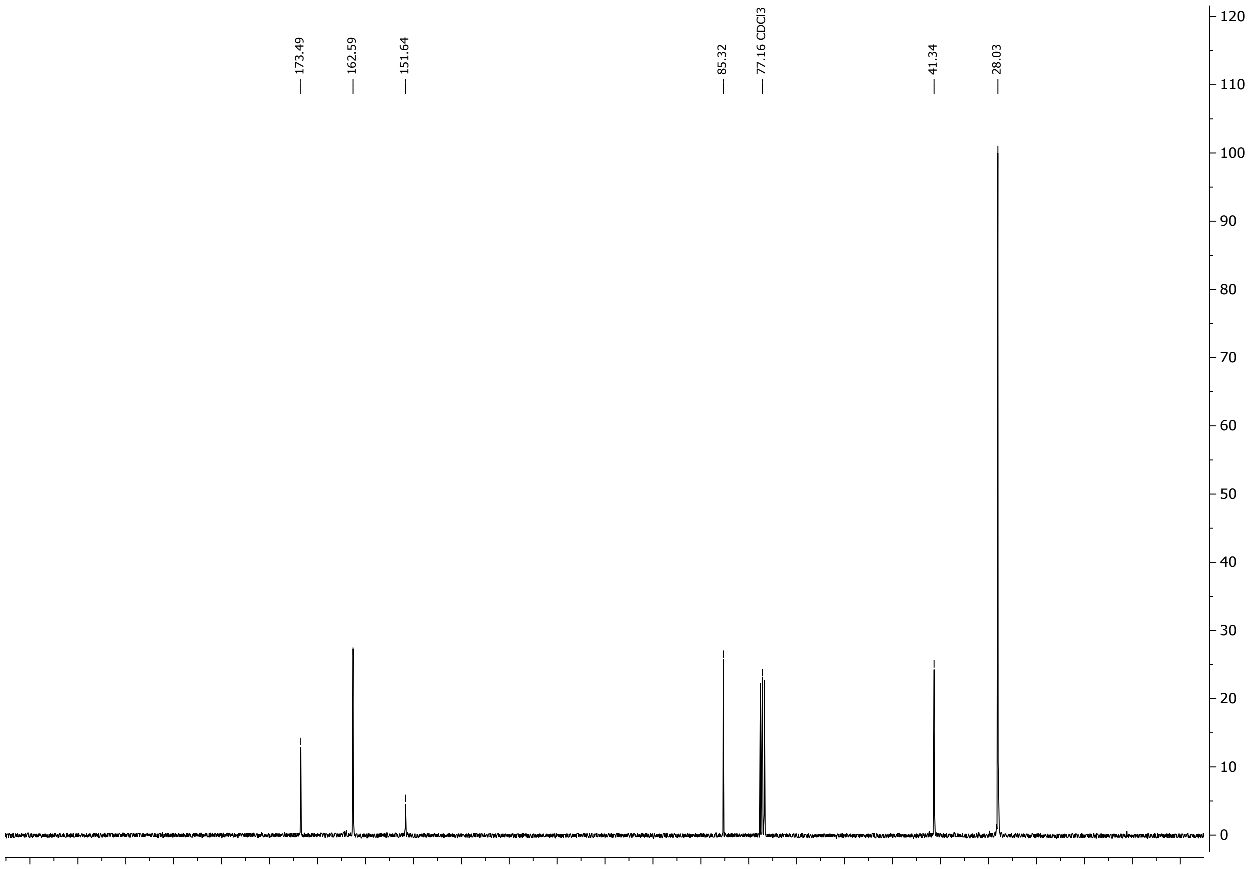

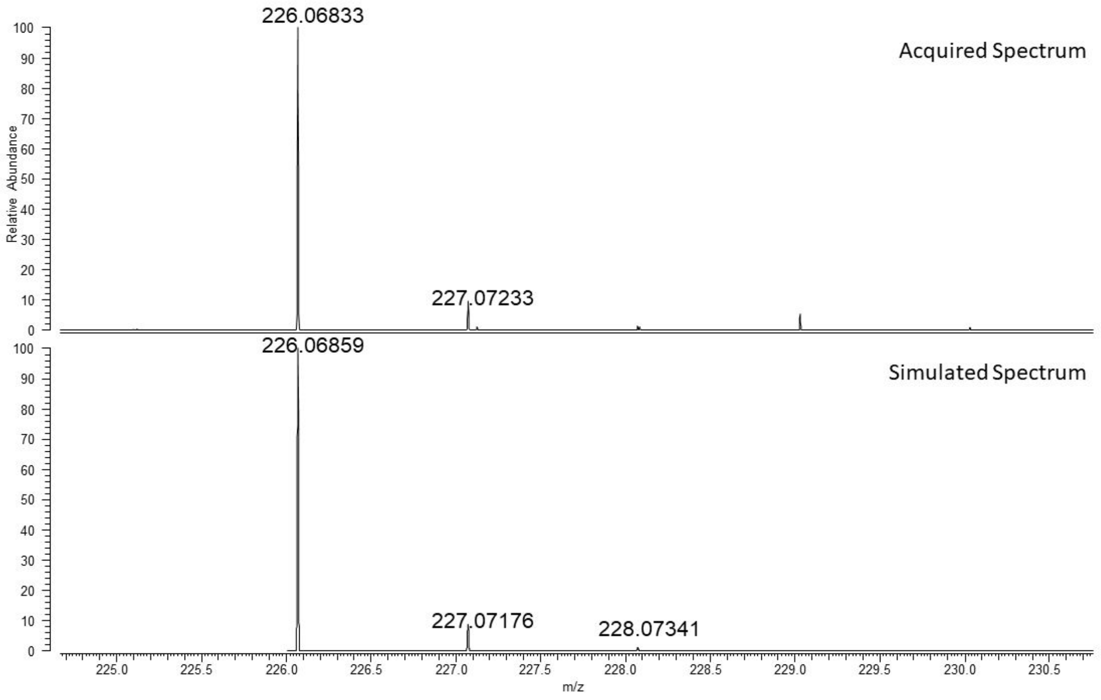
226.06833
Acquired Spectrum
227.07233
226.06859
Simulated Spectrum
227.07176
228.07341
Relative Abundance
m/z

1,4-Dioxepan-5-one (16)

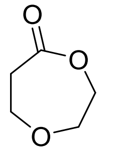

A = ^{1}H-NMR crude product; B = ^{1}H-NMR purified product; C = ^{13}C-NMR.

A

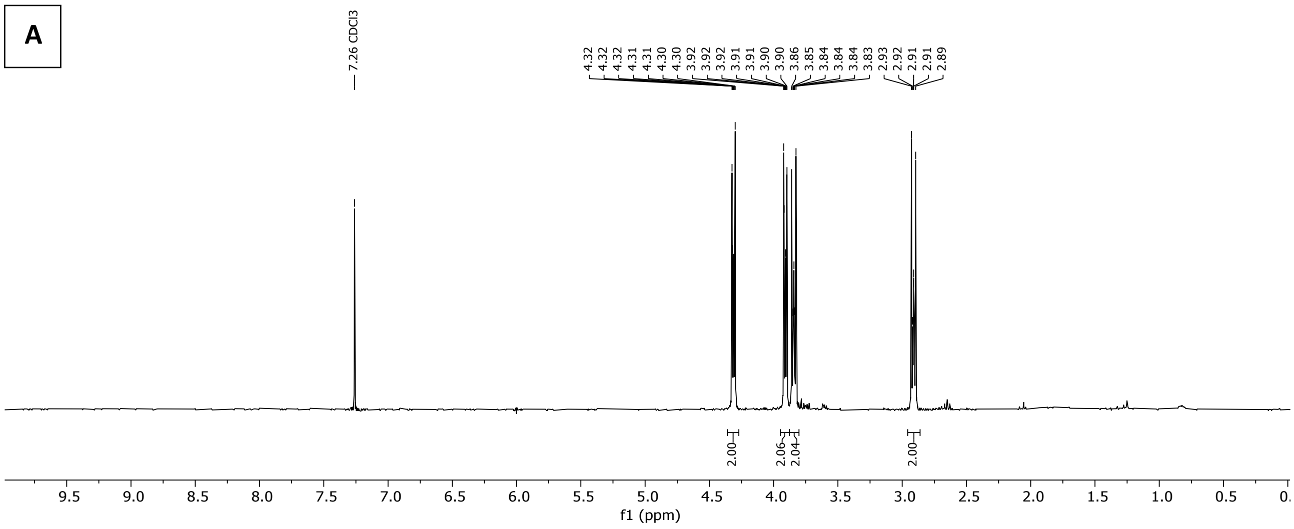

B

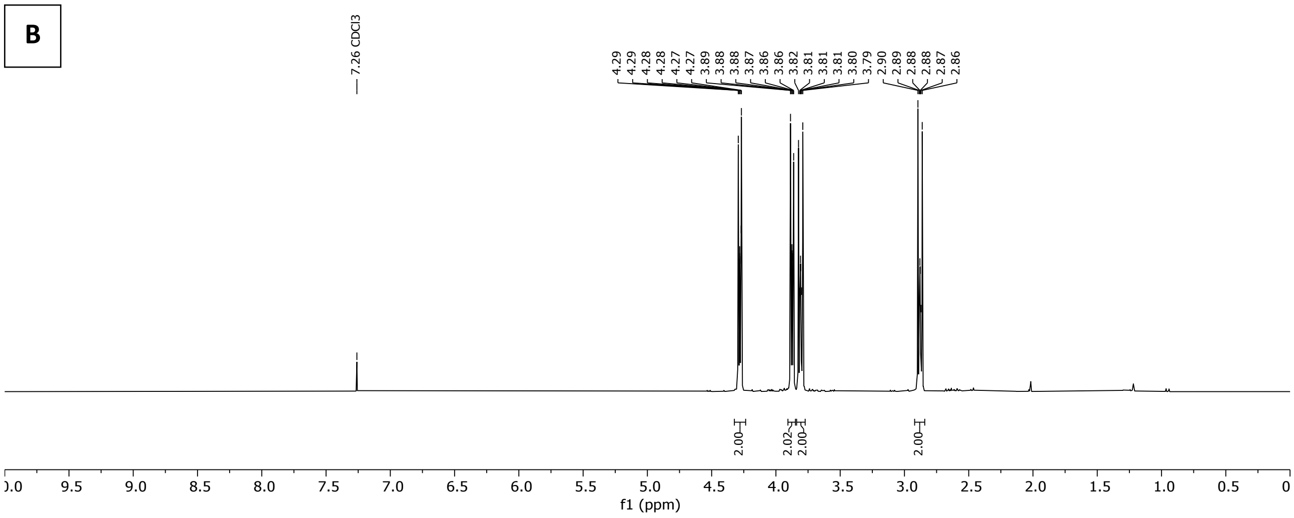

C

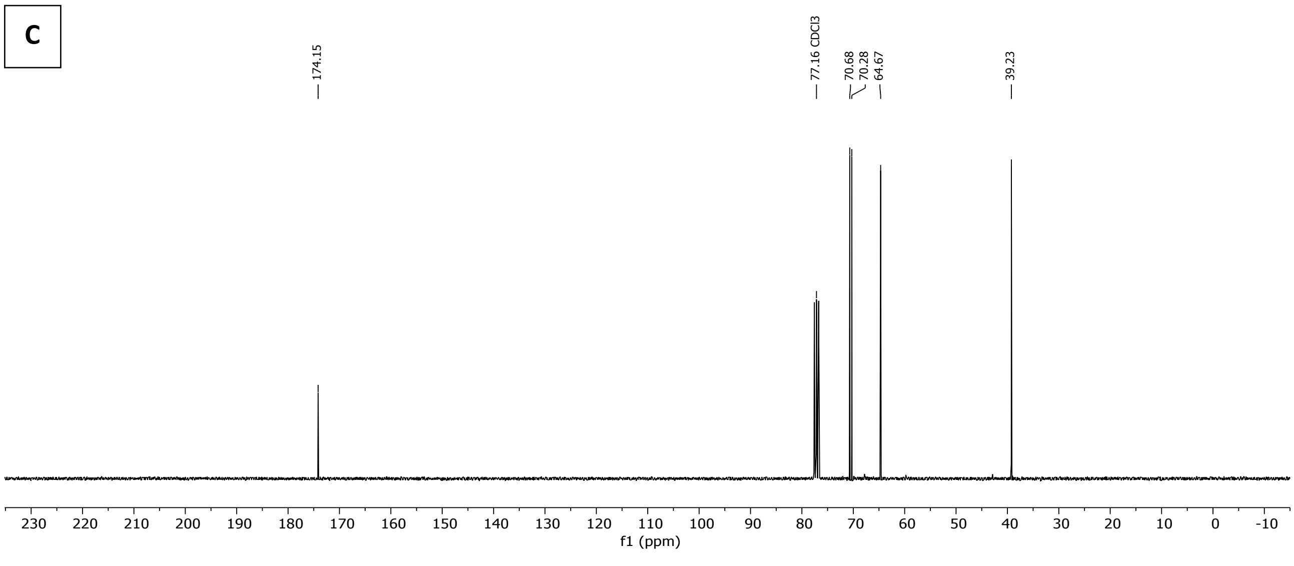

1,3-Dioxepan-4-one (17)

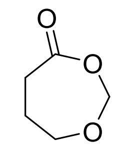

A = ^{1}H-NMR crude product; B = ^{1}H-NMR purified product; C = ^{13}C-NMR.

A

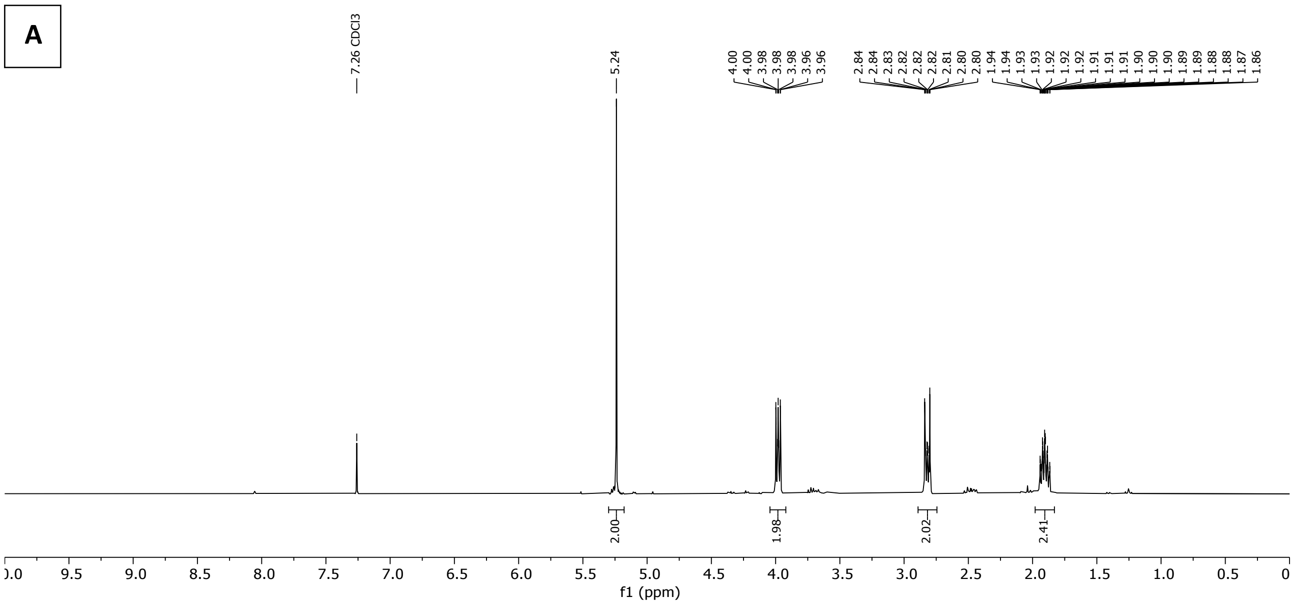

B

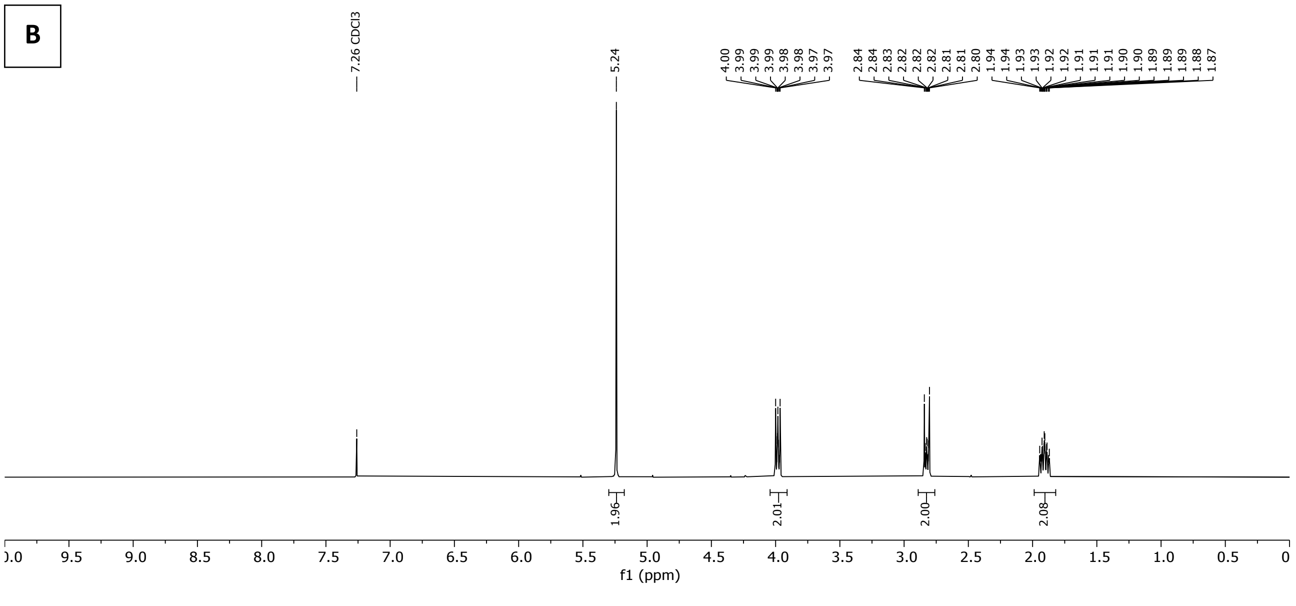

C

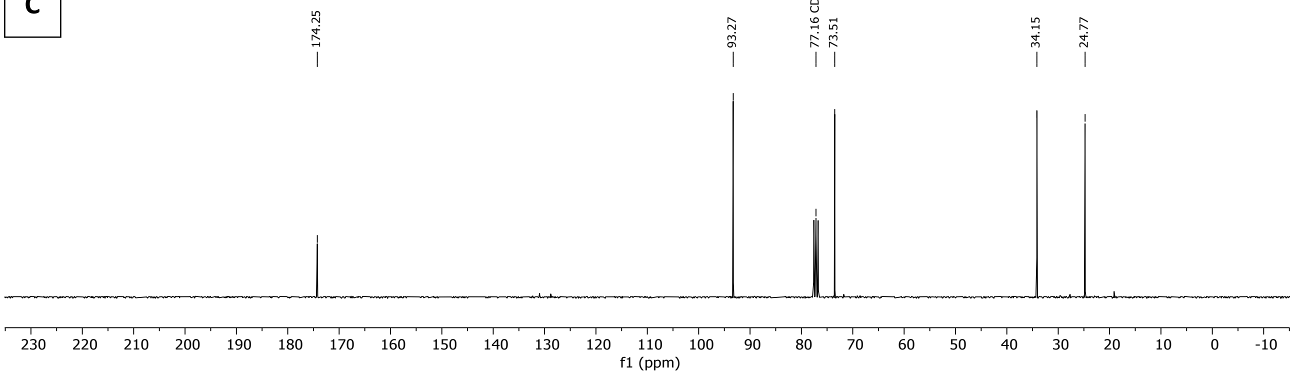

1,3-Dioxan-4-one (18)

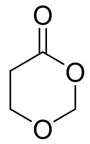

A = ^{1}H-NMR crude product; B = ^{1}H-NMR purified product; C = ^{13}C-NMR.

A

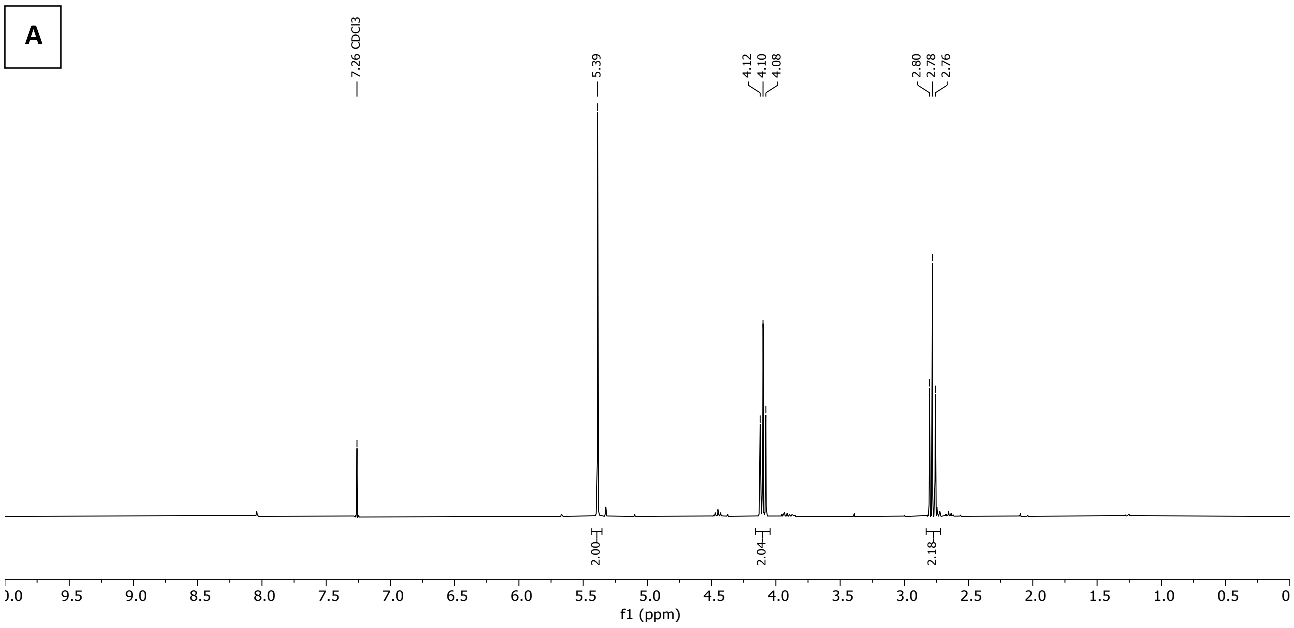

B

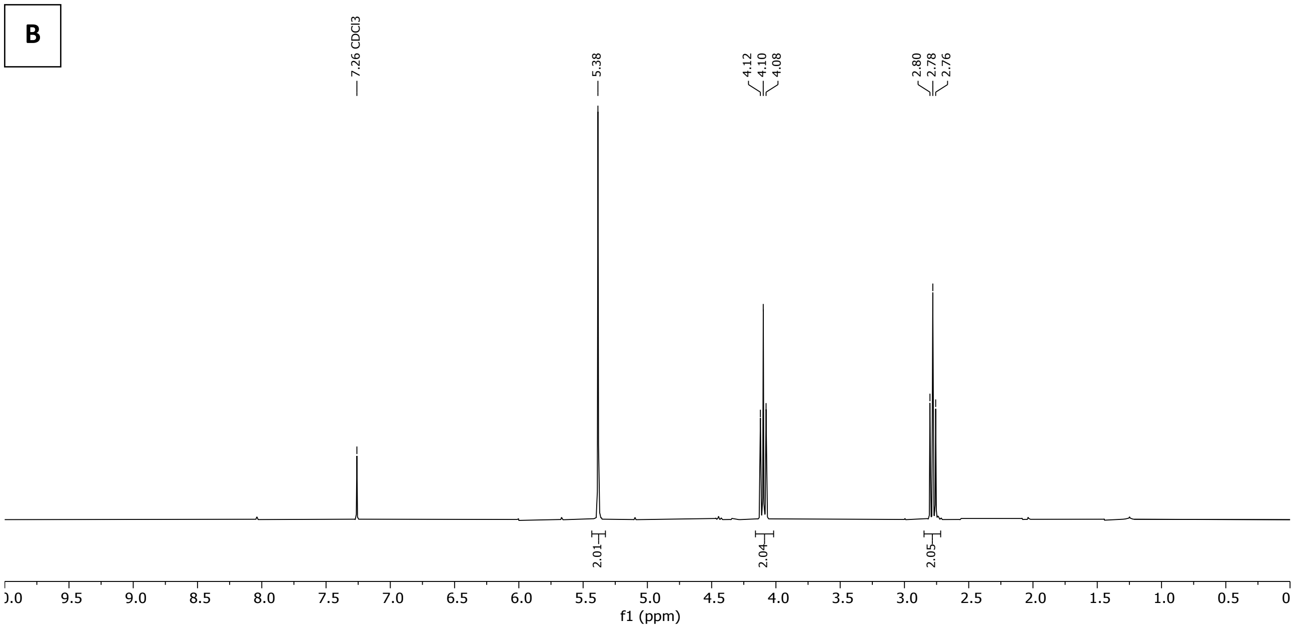

C

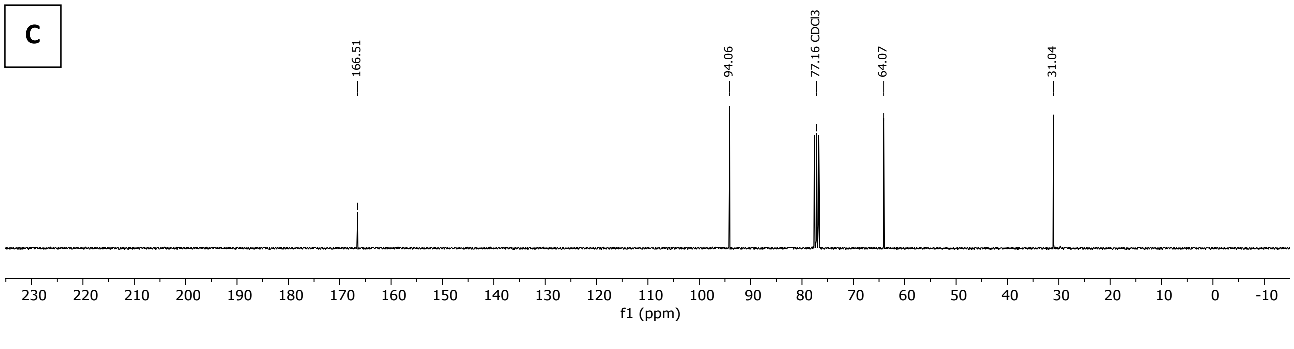

1,3-Dioxolan-4-one (19)

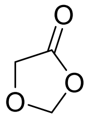

A = ^{1}H-NMR crude product; B = ^{1}H-NMR purified product; C = ^{13}C-NMR.

A

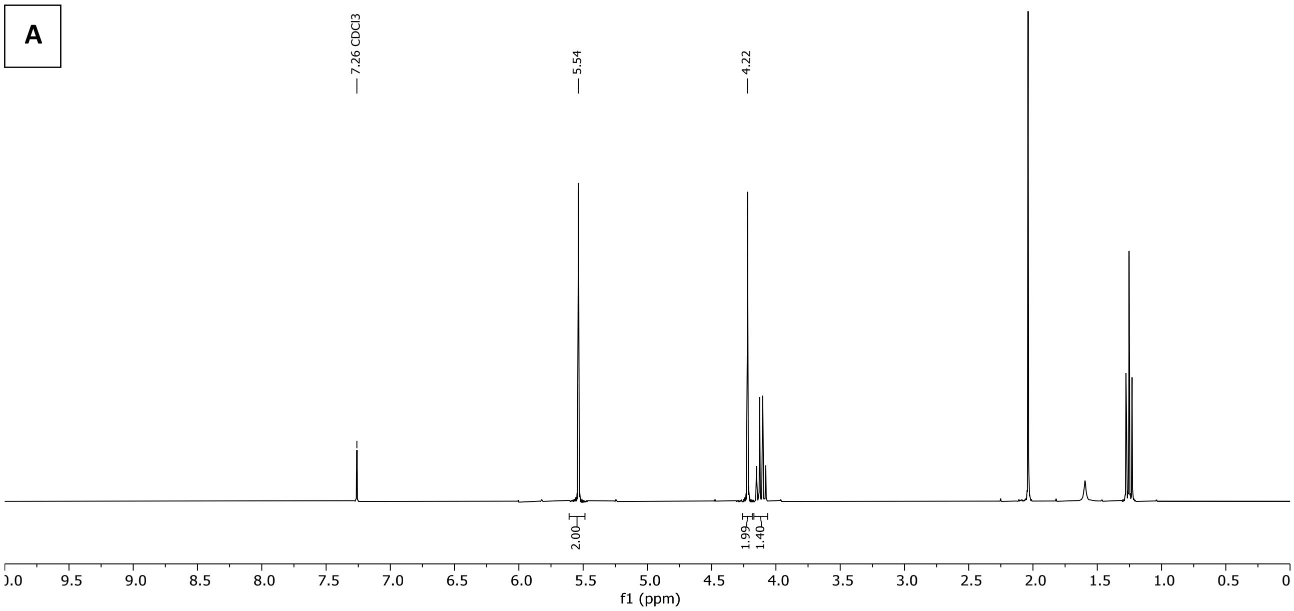

B

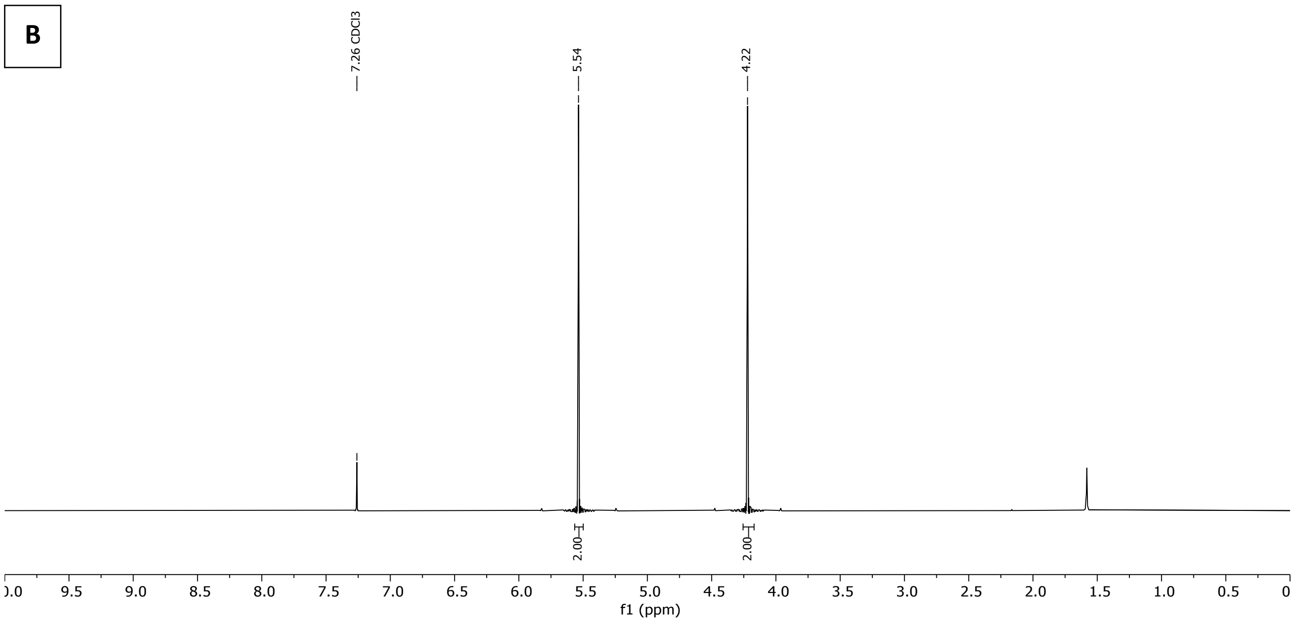

C

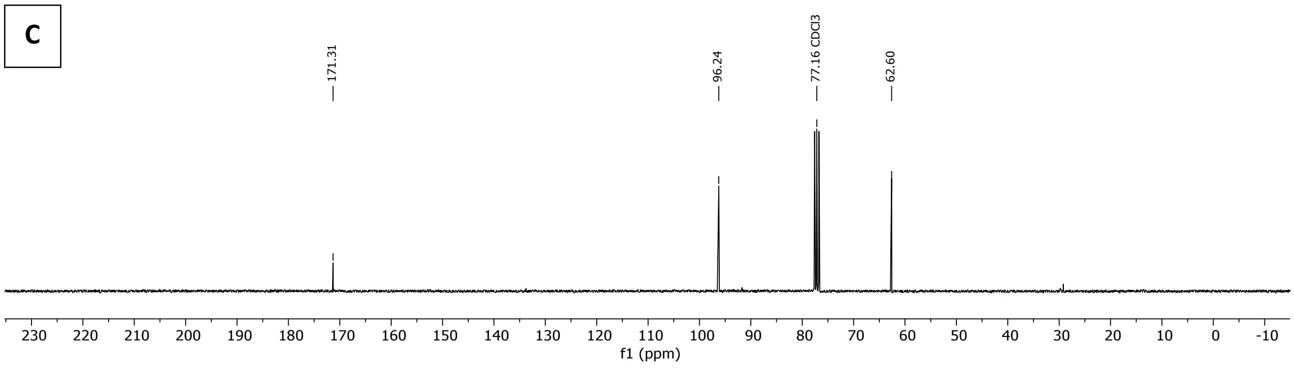

Supplement: Supplementary file 1 — jo3c01513_si_001.pdf [file jo3c01513_si_001.pdf]
